# Sleep in Alzheimer’s disease: a systematic review and meta-analysis of polysomnographic findings

**DOI:** 10.1038/s41398-022-01897-y

**Published:** 2022-04-01

**Authors:** Ye Zhang, Rong Ren, Linghui Yang, Haipeng Zhang, Yuan Shi, Hamid R. Okhravi, Michael V. Vitiello, Larry D. Sanford, Xiangdong Tang

**Affiliations:** 1 Sleep Medicine Center, Department of Respiratory and Critical Care Medicine, Mental Health Center, Translational Neuroscience Center, and State Key Laboratory of Biotherapy, West China Hospital, Sichuan University, Chengdu, China; 2 Center for Integrative Neuroscience and Inflammatory Diseases and Glennan Center for Geriatrics and Gerontology, Department of Internal Medicine, Eastern Virginia Medical School, Norfolk, VA USA; 3 Department of Psychiatry and Behavioral Sciences, University of Washington, Seattle, WA 98195-6560 USA; 4 Sleep Research Laboratory, Center for Integrative Neuroscience and Inflammatory Diseases, Department of Pathology and Anatomy, Eastern Virginia Medical School, Norfolk, VA USA

**Keywords:** Learning and memory, Psychiatric disorders, Biomarkers

## Abstract

Polysomnography (PSG) studies of sleep changes in Alzheimer’s disease (AD) have reported but not fully established the relationship between sleep disturbances and AD. To better detail this relationship, we conducted a systematic review and meta-analysis of reported PSG differences between AD patients and healthy controls. An electronic literature search was conducted in EMBASE, MEDLINE, All EBM databases, CINAHL, and PsycINFO inception to Mar 2021. Twenty-eight studies were identified for systematic review, 24 of which were used for meta-analysis. Meta-analyses revealed significant reductions in total sleep time, sleep efficiency, and percentage of slow-wave sleep (SWS) and rapid eye movement (REM) sleep, and increases in sleep latency, wake time after sleep onset, number of awakenings, and REM latency in AD compared to controls. Importantly, both decreased SWS and REM were significantly associated with the severity of cognitive impairment in AD patients. Alterations in electroencephalogram (EEG) frequency components and sleep spindles were also observed in AD, although the supporting evidence for these changes was limited. Sleep in AD is compromised with increased measures of wake and decreased TST, SWS, and REM sleep relative to controls. AD-related reductions in SWS and REM sleep correlate with the degree of cognitive impairment. Alterations in sleep EEG frequency components such as sleep spindles may be possible biomarkers with relevance for diagnosing AD although their sensitivity and specificity remain to be clearly delineated. AD-related sleep changes are potential targets for early therapeutic intervention aimed at improving sleep and slowing cognitive decline.

## Introduction

Alzheimer’s disease (AD), a neurodegenerative disorder characterized by an impairment in global cognition and progressive memory loss, has been the main cause of dementia and is quickly becoming one of the most lethal, expensive, and burdening diseases in this century [1]. In 2018, AD International estimated a worldwide dementia prevalence of about 50 million people, estimated to triple in 2050, with two-thirds living in middle- and low-income countries [1–3].

Several years before the onset of cognitive impairment (preclinical AD), cerebrospinal fluid (CSF) biomarkers of AD, including amyloid β (Aβ) and tau, begin to pathologically accumulate in the brain [3, 4]. It is believed sleep changes can start in this preclinical stage of AD [5]. Patients at the preclinical stage have more rest-activity rhythm fragmentation, independent of age or sex [6]. More recently in a meta-analysis, including 27 studies, Bubu et al. showed individuals with sleep problems had a 3.78 (95% confidence interval (CI): 2.27–6.30) times higher risk of preclinical AD [7]. It has been suggested that sleep disturbances contribute to cognitive decline and increase the risk of AD by increasing the brain’s Aβ burden [8–10]. Indeed, accumulating evidence indicates that over 45% of AD patients have sleep disturbances [9, 11]. These findings suggest that assessments of sleep disturbances in AD patients may be helpful for identifying targets for preventive and therapeutic approaches to this disease [12–15].

Polysomnography (PSG) is the gold standard method for objectively assessing components of sleep. Evidence suggests that PSG-determined sleep alterations are highly important for understanding the etiology and neurobiology of AD. Recently Ju et al. [16]. found that disrupted slow-wave sleep (SWS) activity, as measured by the change in delta spectral power, significantly increased levels of Aβ, suggesting an important role of SWS in modulating levels of Aβ in the brain. Lucey et al. reported that slow-wave EEG activity (particularly at 1 to 2 Hz) might be helpful in discriminating tau pathology and cognitive decline before or at the earliest stages of AD [17]. Further, rapid eye movement (REM) sleep helps to maintain neuronal homeostasis of the brain; while disturbed REM sleep disrupts neurogenesis and synaptic pruning, and loss of REM sleep has been suggested to result in neurodegeneration [18]. In addition, Liguori et al. reported that disruption in REM sleep was associated with an increase in the levels of CSF orexin in individuals with mild cognitive impairment due to AD [19]. CSF orexin levels have been reported to be positively associated with the levels of tau protein levels in AD patients, suggesting that the dysregulation of the orexinergic system expressed as an increase in CSF orexin levels, is a reflection of more marked and faster tau-mediated neurodegeneration of AD [20].

Many previous reviews discuss sleep changes in AD, but they focus mainly on proposed mechanisms/hypothesis underlying the associations between sleep disturbances and AD (e.g., [21–23]). For PSG features, these reviews typically say that AD patients show significantly altered PSG measured sleep features (e.g., decreased SWS). However, there is a significant problem with the evidence these reviews cite to support these conclusions. These narrative reviews support their conclusions by unsystematically citing PSG studies which show statistically significant PSG parameter changes (e.g., decreased SWS, etc.), while studies with negative findings (e.g., no significant difference in SWS between AD and healthy controls) go unmentioned. (e.g., [24–26]). Thus, the conclusions regarding PSG changes in AD in these reviews are based on selective reporting (ignoring studies with negative findings). Variations in findings across different studies may involve heterogeneity in demographic characteristics (i.e., sex, age, and educational attainment), clinical variability (i.e., disease severity and medication status), and experimental methodology (i.e., PSG recording and scoring methods and the use of adaptation nights). Meta-analytic techniques are useful for resolving discrepancies across studies and for estimating the potential impact of moderators. To our knowledge, no meta-analytic study on PSG measured sleep in AD has been conducted to date. To fill this gap, we systematically reviewed previous case-control studies and used meta-analytic procedures to identify the pooled effect sizes for the differences in PSG measured sleep between AD patients and healthy controls where possible. We also explored potential moderators of the PSG changes in AD patients compared with healthy controls.

## Methods

### Protocol and registration

We registered the protocol for this study (PROSPERO ID: CRD42021240066) according to the preferred reporting criteria for systematic reviews and meta-analyses statements [27].

### Inclusion and exclusion criteria

To explore the nighttime PSG differences between AD patients and healthy controls, we included only case-control studies that made comparisons between these groups. The included studies were selected to meet the following criteria:

1. The patients met diagnostic criteria for AD according to recognized criteria from the National Institute of Neurological and Communicative Disorders and Stroke-Alzheimer’s Disease and Related Disorders Association workgroup [28], the National Institute on Aging-Alzheimer’s Association workgroup [29], or the Diagnostic and Statistical Manual of Mental Disorders [30]. When the diagnostic criteria for AD were not specified, studies in which AD status was determined by physicians’ clinical assessments (i.e., patients having acquired global impairment of intellect, memory, and personality) and neuroimaging findings (i.e., presence of diffuse atrophy in the brain but without cerebrovascular accident or other focal intracranial pathologic changes.) were also included.
2. The included studies included a healthy control group.
3. The studies reported differences in some PSG measured nighttime sleep parameters between AD patients and healthy controls (the PSG parameters of interest are listed below in the section on “Data collection process”).
4. The studies were published in English in peer-reviewed journals.
5. If the same participants were used in more than one study, then only the dataset with the most relevant information was used to avoid data duplication.

By screening titles, abstracts, and full text, we excluded: (1) animal studies; (2) editorials, case reports, case series, guidelines, comments, statements, and review papers; (3) studies unrelated to AD; (4) studies not including healthy controls or not clearly clarifying whether their controls are healthy controls; (5) studies not reporting PSG data in AD patients or healthy controls; and (6) studies containing no information on outcomes of interest.

### Information sources, search, and study selection

We searched MEDLINE via OVID; EMBASE via OVID; all EBM databases via OVID; CINAHL via EBSCO; and PsycINFO via EBSCO. The following terms were searched for in the abstract or title: (“Alzheimer Disease” OR “Alzheimer’s disease”) AND (“polysomnogra*” OR “PSG” OR “sleep architect*” OR “sleep monit*” OR “sleep stage*” OR “electroencephalogra*” OR “EEG”). The detailed search strategies used in each literature database are provided in Tables S1–S5. The reference lists of all primary studies were also screened for additional references. We performed the literature search on March 6, 2021. Two reviewers (RR and YZ) independently selected relevant papers. Discrepancies were resolved by discussion with the senior author (XT). If the PSG data of multiple AD groups (e.g., mild and moderate AD patients) were reported and separately compared with controls in a study, this approach was carried over into the current meta-analysis.

### Data collection process and quality assessment of included studies

RR and YZ independently extracted the data from the reviewed studies using a pre-designed form. The extracted data were entered by YZ and verified by RR and YZ. Discrepancies were resolved by discussion with XT. The PSG variables examined in our systematic review include sleep macrostructure: total sleep time (TST), sleep efficiency (SE), wake time after sleep onset (WASO), sleep latency (SL), and percentage of sleep stages N1, N2, N3 and REM sleep, REM latency (REML), and REM density (REMD). In the American Academy of Sleep Medicine (AASM) scoring rules, N3 represents SWS and replaces sleep stages 3 and 4 in the Rechtschaffen and Kales (R&K) nomenclature [31]. Thus, the data for stages 3 and 4 in the included studies were also extracted for estimating SWS. Other PSG variables include periodic limb movement during sleep (PLMS) index and sleep microstructure parameters: cyclic alternating pattern (CAP) parameters, power spectral analysis (PSA) data (i.e., alpha, beta, delta, theta, and gamma frequency activity), and sleep spindle data. We also extracted demographic, clinical, and methodological variables, including the number of participants and their mean age, sex (male percentage), educational attainment (years), Mini-Mental State Examination (MMSE) scores, whether the patients were free of medications impacting sleep (Yes vs. No), PSG scoring methods (R&K vs. AASM), and use of an adaptation night (Yes vs. No). RR and YZ independently assessed the risk of bias of the included studies by using the adapted version of the National Institute for Health and Care Excellence (NICE) checklist [32], with discrepancies resolved in discussion with XT.

### Statistical analysis

To calculate the pooled effect sizes (standardized mean difference (SMD)) for the PSG changes in AD patients compared with controls, the sample size, mean, and standard deviation for the two groups were entered. For the estimation of global effect-size for each PSG parameter, the I^2^ and Q statistics were calculated to examine the presence and magnitude of heterogeneity and to inform on the degree of overlap between the 95% CIs of included studies. The random-effects model was applied to obtain relatively conservative meta-analysis findings. The Egger regression method [33] was used to examine publication bias, with *p* values of <0.05 suggesting the presence of bias. Duval and Tweedie’s trim and fill test was conducted to get the adjusted effect sizes when publication bias was present [34]. A meta-regression or subgroup analysis (depending on whether the potential moderators were continuous or categorical variables) was conducted to determine the potential factors that could moderate heterogeneity between studies. All analysis in our meta-analysis was done using Comprehensive Meta-Analysis software.

## Results

### Study selection

**Fig. 1:**
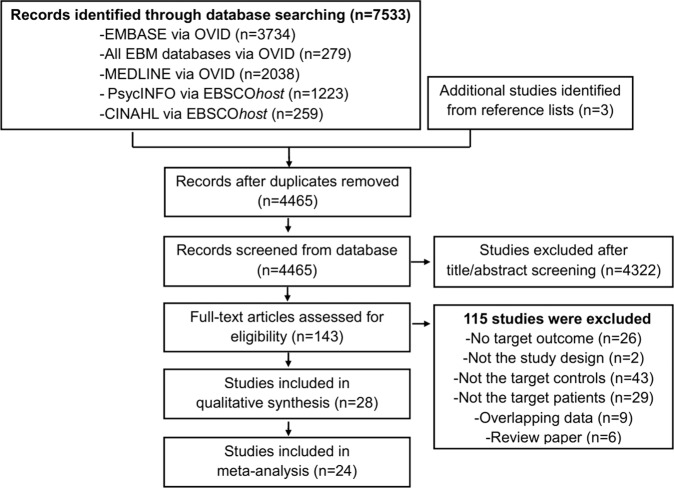
Flow chart used for the identification of eligible studies.

Our search yielded 7533 publications (Fig. 1). After removing the duplicates, the title/abstract of the remaining 4465 articles were screened. A total of 143 studies were selected for full-text review. Of these, 28 articles [24–26, 35–59] were found to meet inclusion criteria for the systematic review (Table 1), and 24 of the 28 studies were included in our meta-analysis. The excluded studies with reasons for their exclusion are shown in Table S6.

**Table 1:** Study characteristics.

Study	Sample size	Percentage male	Mean age	Education (years)	MMSE	Free of medications impacting sleep	Adaptation	PSG scoring methods
Bonakis et al. [24]	17 AD	52.9%	69.0 ± 9.9	NR	17.9 ± 5.63	Yes	Yes	AASM
	20 controls	60%	70.2 ± 12.5	NR			Yes	AASM
Bonanni et al. [35]	11 AD (mild)	45.5%	65.6 ± 7.4	NR	22.1 ± 1.4	Yes	Yes	R&K
	9 AD (moderate)	44.4%	64 ± 8.7	NR	13.7 ± 3.3	Yes	Yes	R&K
	12 controls	58.3%	61.1 ± 5.1	NR	28.4 ± 1.4		Yes	R&K
Brunetti et al. [36]	47 AD	40%	73.57 ± 6.71	8.89 ± 4.91	18.38 ± 4.70	Yes	No	R&K
	44 controls	62%	70.64 ± 6.73	11.37 ± 4.64	27.54 ± 1.65		No	R&K
Chen et al. [37]	22 AD (mild)	50%	70.8 ± 10.7	12.3 ± 3.4	22.0 ± 2.3	Yes	Yes	R&K
	21 AD (moderate)	42.9%	69.2 ± 11.5	11.0 ± 3.2	15.5 ± 4.4	Yes	Yes	R&K
	22 controls	50%	66.9 ± 6.7	10.3 ± 3.2	30 ± 0		Yes	R&K
Dykierek et al. [38]	35 AD	45.7%	62.1 ± 8.9	NR	19.5 ± 5.2	Yes	Yes	R&K
	42 controls	52.4%	64.4 ± 7.5	NR	29.2 ± 1.0		Yes	R&K
Gagnon et al. [39]	15 AD	46.7%	70.2 ± 5.6	NR	NR	Yes	No	R&K
	15 controls	73.3%	67.9 ± 5.4	NR	NR		No	R&K
Gorgoni et al. [40]	15 AD	33.3%	70.80 ± 9.30	9.4 ± 5.77	16.07 ± 4.26	Yes	No	R&K
	15 controls	66.7%	70.80 ± 6.31	11.8 ± 4.80	29.07 ± 1.05		No	R&K
Liguori et al. [43]	20 AD	35%	66.3 ± 4.18	NR	21.4 ± 1.93	Yes	Yes	AASM
	15 controls	53.3%	63.8 ± 8.46	NR	29.6 ± 1.47		Yes	AASM
Liguori et al. [44]	56 AD (mild)	37.5%	69.93 ± 7.27	NR	24.45 ± 1.85	Yes	Yes	AASM
	48 AD (moderate-severe)	39.6%	71.71 ± 7.19	NR	15.40 ± 3.21	Yes	Yes	AASM
	41 controls	48%	67.17 ± 9.83	NR	29.20 ± 0.90		Yes	AASM
Liu et al. [45]	30 AD	20%	75.77 ± 5.69	4.63 ± 3.99	21.23 ± 1.65	Yes	Yes	AASM
	30 controls	26.7%	75.13 ± 6.32	5.27 ± 4.43	29.03 ± 1.16		Yes	AASM
Rauchs et al. [25]	14 AD	35.7%	76.9 ± 4.1	NR	24.9 ± 2	Yes	Yes	R&K
	14 controls	35.7%	75.1 ± 4.6	NR	29.4 ± 0.9		Yes	R&K
Reda et al. [51]	20 AD	35%	72 ± 8.59	9.65 ± 5.23	16.40 ± 4.70	Yes	No	R&K
	20 controls	60%	70.35 ± 6.26	11.60 ± 5	28.75 ± 1.30		No	R&K
Yin et al. [58]	123 AD	36.6%	72.1 ± 7.2	11.4 ± 3.3	19.7 ± 5.4	Yes	Yes	R&K
	120 controls	34.2%	70.9 ± 7.3	11.6 ± 2.8	28.3 ± 1.5		Yes	R&K
Tseng et al. [55]	5 AD	40%	76.40 ± 3.51	NR	19.80 ± 1.79	Yes	No	R&K
	9 controls	66.7%	76.89 ± 7.51	NR	30		No	R&K
Reynolds et al. [54]	25 AD	28%	70.4 ± 8.3	11.4 ± 4.0	NR	Yes	Yes	R&K
	25 controls	32%	69.0 ± 5.0	14.5 ± 4.1	NR		Yes	R&K
Reynolds et al. [53]	49 AD	20.4%	72.8 ± 8.0	NR	16.5 ± 6.0	Yes	Yes	R&K
	77 controls	44.2%	69.3 ± 6.4	NR	29.4 ± 0.8		Yes	R&K
Reynolds et al. [52]	22 AD	31.8%	70.9 ± 8.1	NR	NR	Yes	Yes	R&K
	24 controls	33.3%	69.5 ± 4.5	NR	NR		Yes	R&K
Prinz et al. [50]	18 AD (Mild)	50%	67.8 ± 9.46	15.2 ± 3.52	16.7 ± 5.52	NR	Yes	R&K
	16 AD (Moderate)	63%	70.2 ± 6.16	15. 1 ± 3.16	5.4 ± 3.6	NR	Yes	R&K
	10 AD (Severe)	100%	72.8 ± 10.97	12.6 ± 2.45	1.3 ± 1.83	NR	Yes	R&K
	22 controls	50%	69 ± 6.43	14.2 ± 5.47	29.6 ± 0.86		Yes	R&K
Prinz et al. [49]	10 AD	100%	73.30 ± 11.4	NR	3.30 ± 6.52	Yes	Yes	R&K
	11 controls	100%	72.18 ± 10.5	NR	NR		Yes	R&K
Petit et al. [48]	8 AD	50%	60.6	NR	NR	Yes	Yes	R&K
	8 controls	50%	58.3	NR	NR		Yes	R&K
Vitiello et al. [57]	44 AD	45%	70.7 ± 7.5	13.8 ± 3.1	22.7 ± 2.9	Yes	Yes	R&K
	45 controls	44%	66.8 ± 6.7	14.3 ± 3.5	29.7 ± 0.6		Yes	R&K
Vitiello et al. [56]	9 AD (Mild)	44.4%	65.7 ± 3.1	NR	18.9 ± 1.5	Yes	Yes	R&K
	9 AD (Moderate)	55.6%	70.0 ± 2.0	NR	6.2 ± 1.4	Yes	Yes	R&K
	9 AD (Severe)	100%	73.0 ± 3.9	NR	3.3 ± 2.3	Yes	Yes	R&K
	9 controls	22.2%	65.6 ± 1.9	NR	29.7 ± 0.3		Yes	R&K
Martin et al. [26]	8 AD	75%	NR	NR	NR	Yes	Yes	R&K
	9 controls	66.7%	NR	NR	NR		Yes	R&K
Maestri et al. [46]	11 AD	36.4%	72.7 ± 5.9	NR	21.2 ± 0.8	Yes	No	AASM
	11 controls	54.5%	69.2 ± 12.6	NR	29.3 ± 1.0		No	AASM
Hot et al. [42]	14 AD	50%	76.7 ± 3.8	NR	24.8 ± 2.4	Yes	Yes	R&K
	14 controls	42.9%	76.7 ± 4.1	NR	29.4 ± 1		Yes	R&K
Hassainia et al. [41]	27 AD	44.4%	70.1	NR	20.2	Yes	No	R&K
	25 controls	52%	67.8	NR	28.6		No	R&K
Montplaisir et al. [47]	10 AD	NR	60.6	NR	20.6 ± 5.3	NR	Yes	R&K
	10 controls	NR	58.3	NR	29.3 ± 1.0		Yes	R&K
Prinz et al. [59]	20 AD (male)	100%	70 ± 7	14 ± 3	23 ± 3	Yes	Yes	R&K
	19 AD (female)	0	72 ± 8	14 ± 3	22 ± 3	Yes	Yes	R&K
	17 controls (male)	100%	66 ± 5	15 ± 4	30 ± 0		Yes	R&K
	26 controls (female)	0	68 ± 8	14 ± 3	30 ± 1		Yes	R&K

*AASM* American Academy of Sleep Medicine, *AD* Alzheimer’s disease, *MMSE* mini-mental state examination, *NR* not reported, *R&K* Rechtschaffen and Kales.

### Description of the included studies

As shown in Table 1, the sample sizes of the 28 studies ranged from 16 participants (eight AD patients and eight controls) [48] to 243 participants (123 AD patients and 120 controls) [58]. The mean age of AD patients and controls ranged from 58.3 to 75.8 y (reported in 27 studies). Males as percentages of AD patients and controls ranged from 0–100% (reported in 27 studies). Five studies [24, 43–46] used AASM PSG criteria and the other 23 studies used R&K rules. For PSG adaptation nights, seven studies [36, 39–41, 46, 51, 55] did not include an adaptation night and the other 21 studies included an adaptation night. Among the 28 studies, two studies [47, 50] did not report whether they excluded AD patients who used medication impacting sleep, the other 26 studies clearly stated that their AD patients were drug naïve or had a washout period for medications impacting sleep before PSG examinations. For quality assessments of these studies (Table S7), no study addressed all ten aspects of the NICE checklist. For instance, no study reported a participation rate nor compared participants vs. non-participants. Furthermore, 15 studies did not report whether the same exclusion criteria were used for both cases and controls.

### Meta-analysis

In the whole sample, the meta-analysis revealed significantly decreased TST, SE, SWS, and REM sleep percentage, and REMD, and significantly increased N1 percentage, SL, WASO, number of awakenings, and REML in AD patients compared with controls (Table 2). There were no significant differences in N2 percentage and PLMS index between AD patients and controls (*p* > 0.05).

**Table 2:** Summary of meta-analysis comparing AD patients and controls.

	No. of comparisons	No. of AD/controls	Means of AD	Means of controls	SMD (95%CI)	Q	I^2^
N1%	19	504/495	18.410	12.707	0.820 (0.369 to 1.271)***	186.036***	90.324
N2%	20	551/539	57.707	58.310	0.092 (−0.233 to 0.416)	118.626***	83.983
Number of awakenings	15	383/415	16.147	13.547	0.551 (0.246 to 0.855)***	53.118***	73.644
PLMS index	5	80/88	2.422	4.088	−0.158 (−0.501 to 0.185)	4.929	18.855
REM%	25	668/711	13.965	17.673	−0.767 (−1.142 to −0.391)***	241.492***	90.062
REMD	7	129/165	–	–	−0.286 (−0.545 to −0.028)*	6.644	9.698
REML	23	609/646	111.404	92.814	0.352 (0.127 to 0.578)**	74.566***	70.496
SE	20	579/594	71.331	80.643	−0.962 (−1.358 to −0.567)***	174.611***	89.119
SL	20	588/610	22.629	14.894	0.451 (0.292 to 0.609)***	30.177*	37.038
SWS%	25	644/659	4.722	9.956	−0.861 (−1.142 to −0.580)***	125.750***	80.915
TST	23	644/660	330.904	369.504	−0.596 (−0.856 to −0.335)***	102.455***	78.527
WASO	16	540/541	104.419	73.799	0.739 (0.375 to 1.103)***	112.309***	86.644

Means for REMD were not calculated because definitions and algorithms of REMD varied across studies.

*%* percentage, *AD* Alzheimer’s disease, *Q* Cochran’s Q statistic, *HCs* healthy controls, *PLMS* periodic limb movement during sleep, *REM* rapid eye movement sleep, *REMD* REM density, *REML* REM latency, *SWS* slow-wave sleep, *SE* sleep efficiency, *SL* sleep latency, *SMD* standardized mean difference, *TST* total sleep time, *WASO* wake time after sleep onset.

*p < 0.05, **p < 0.01, ***p < 0.001.

For these findings, the Egger test failed to detect any publication bias (Figs. S1–S11), although it should be noted that the Egger test for the differences in PLMS index and REMD could not be performed because of limited available data.

### Moderator analysis

As shown in Table 3, a meta-regression analysis revealed that decreased percentage of male AD patients (*p* = 0.01) and increased age of AD patients (*p* = 0.008) across different studies were significantly associated with increased SL in AD patients compared with controls.

**Table 3:** Moderator analyses.

Moderators		TST	WASO	Number of awakenings	SE	SL	N1%	N2%	SWS%	REM%	REML	REMD
**Sex (male%)**	No. of comparisons	22	16	15	20	19	18	19	24	24	22	6
	No. of AD/Controls	634/650	540/541	383/415	579/594	578/600	494/485	541/529	634/649	658/701	599/636	119/155
	Point estimate	0.477	0.793	1.354	1.651	−1.290	−3.561	−1.189	0.378	−0.557	0.202	−1.077
	SE	1.319	2.410	0.991	1.836	0.502	2.168	1.626	0.989	1.270	0.743	0.550
	P	0.718	0.742	0.172	0.369	0.010	0.100	0.464	0.702	0.661	0.786	0.0503
**Mean age**	No. of comparisons	22	16	15	19	19	18	19	24	24	22	6
	No. of AD/Controls	636/651	540/541	383/415	571/585	580/601	496/486	543/530	636/650	660/702	601/637	121/156
	Point estimate	−0.019	−0.071	−0.023	−0.001	0.048	0.041	0.004	−0.002	0.028	0.004	0.036
	SE	0.033	0.056	0.041	0.053	0.018	0.057	0.041	0.037	0.049	0.027	0.024
	P	0.567	0.206	0.575	0.983	0.008	0.470	0.922	0.952	0.570	0.882	0.133
**Education (years)**	No. of comparisons	8	8	9	7	6	7	8	12	11	8	–
	No. of AD/Controls	322/318	322/318	289/310	278/273	287283	256/254	303/298	391/409	366/384	287/304	–
	Point estimate	0.053	0.019	0.151	0.030	−0.035	−0.473	−0.200	0.080	0.023	0.032	–
	SE	0.088	0.079	0.086	0.098	0.038	0.059	0.119	0.054	0.078	0.046	–
	P	0.542	0.811	0.079	0.762	0.359	<0.001	0.094	0.138	0.768	0.492	–
**MMSE score**	No. of comparisons	21	16	15	18	18	16	17	22	23	21	6
	No. of AD/Controls	621/636	540/541	383/415	556/570	565/586	456/446	503/490	596/610	645/687	586/622	121/156
	Point estimate	0.044	0.023	−0.022	−0.027	0.027	−0.011	0.011	0.049	0.067	−0.026	0.011
	SE	0.045	0.057	0.025	0.067	0.021	0.080	0.054	0.028	0.037	0.022	0.030
	P	0.330	0.681	0.370	0.693	0.202	0.888	0.842	0.075	0.071	0.229	0.714
**Medication free**												
Not reported	No. of comparisons	1	–	3	–	1	1	1	4	4	4	1
	No. of AD/Controls	10/10	–	44/66	–	10/10	10/10	10/10	54/76	54/76	54/76	10/10
	SMD	−0.062	–	0.895***	–	−0.240	0.601	0.277	−1.100***	−1.375***	0.713***	−0.749
	Q	0	–	0.010	–	0	0	0	3.198	10.498*	2.654	0
	I^2^	0	–	0	–	0	0	0	6.184	71.424	0	0
Yes	No. of comparisons	22	16	12	20	19	18	19	21	21	19	6
	No. of AD/Controls	634/650	540/541	339/349	579/594	578/600	494/485	541/529	590/583	614/635	555/570	119/155
	SMD	−0.616***	0.739***	0.471**	−0.962***	0.471***	0.831**	0.084	−0.818***	−0.654**	0.290*	−0.245
	Q	101.331***	112.309***	47.307***	174.611***	27.648	186.036***	118.443***	120.355***	216.640***	66.595***	5.472
	I^2^	79.216	86.644	76.748	89.119	34.895	90.862	84.803	83.383	90.768	72.971	8.633
Between-group difference	Q	1.404	–	2.415	–	2.431	0.199	0.162	1.196	2.737	3.531	1.094
	P	0.236	–	0.120	–	0.119	0.656	0.688	0.274	0.098	0.060	0.296
**Adaptation night**												
No	No. of comparisons	6	4	3	5	3	4	5	6	6	4	–
	No. of AD/Controls	113/114	93/90	46/46	102/103	67/68	61/61	108/105	113/114	113/114	78/79	–
	SMD	−0.526**	0.018	0.054	−0.495**	0.657***	0.870***	−0.006	−0.635**	−0.415	0.164	–
	Q	7.131	0.034	3.998	1.017	0.777	4.303	15.965**	12.638*	17.452**	5.657	–
	I^2^	29.880	0	49.979	0	0	30.280	74.946	60.438	71.350	46.972	–
Yes	No. of comparisons	17	12	12	15	17	15	15	19	19	19	7
	No. of AD/Controls	531/546	447/451	337/369	477/491	521/542	443/434	443/434	531/545	555/597	531/567	129/165
	SMD	−0.599***	0.974***	0.663***	−1.091***	0.413***	0.789**	0.119	−0.925***	−0.862***	0.385**	−0.286*
	Q	95.029***	93.838***	44.358***	168.870***	28.124*	179.947***	98.831***	109.629***	218.276***	67.292***	6.644
	I^2^	83.163	88.278	75.202	91.710	43.110	92.220	85.834	83.581	91.754	73.251	9.698
Between-group difference	Q	0.092	12.830	3.045	4.037	1.491	0.051	0.121	0.976	1.524	0.603	–
	P	0.792	<0.001	0.081	0.045	0.222	0.822	0.728	0.323	0.217	0.438	–
**PSG scoring methods**												
AASM	No. of comparisons	6	5	1	5	4	5	5	5	5	6	
	No. of AD/Controls	182/158	165/138	11/11	171/147	151/132	162/143	162/143	162/143	162/143	182/158	–
	SMD	−1.108***	1.386***	0.774	−1.996***	0.488**	2.156***	0.456	−1.699**	−2.170***	0.587*	–
	Q	26.862***	26.104***	0	59.169***	4.833	14.677**	65.047***	49.543***	35.402***	18.508**	–
	I^2^	81.386	84.677	0	93.240	37.924	72.747	93.851	91.926	88.701	72.984	–
R&K	No. of comparisons	17	11	14	15	16	14	15	20	20	17	7
	No. of AD/Controls	462/502	375/403	372/404	408/447	437478	342/352	389/396	482/516	506/568	427/488	129/165
	SMD	−0.398**	0.435**	0.539**	−0.597***	0.437***	0.301*	−0.044	−0.650***	−0.385**	0.250*	−0.286*
	Q	41.806***	31.063**	52.508***	32.323**	25.181*	25.200*	44.029***	39.866**	62.067***	39.630**	6.644
	I^2^	61.728	67.808	75.242	56.687	40.430	48.412	68.203	52.340	69.388	59.626	9.698
Between-group difference	Q	5.190	6.250	0.249	5.964	0.074	34.444	0.843	4.129	14.221	1.719	–
	P	0.023	0.012	0.618	0.015	0.786	<0.001	0.358	0.042	<0.001	0.190	–

A meta-regression or subgroup analysis (depending on whether the potential moderators were continuous or categorical variables) was conducted to determine the potential factors that could moderate heterogeneity between studies. Of which, the effects of sex, age, education, and MMSE score were explored by using a meta-regression analysis, while the effects of whether taking medication impacts sleep (Yes vs. No), adaptation night (Yes vs. No), and PSG scoring methods (AASM vs. R&K) were explored by using subgroup analysis.

*AASM* American Academy of Sleep Medicine, *AD* Alzheimer’s disease, *AHI* apnea-hypopnea index, *Q* Cochran’s Q statistic, *MMSE* mini-mental state examination, *PSG* polysomnography, *REM* rapid eye movement sleep, *REMD* REM density, *REML* REM latency, *R&K* Rechtschaffen and Kales, *SMD* standardized mean difference, *SWS* slow-wave sleep, *SE* sleep efficiency, *SL* sleep latency, *TST* total sleep time, *WASO* wake time after sleep onset.

*p < 0.05, **p < 0 .01, ***p < 0.001.

To exclude the potential effects of medication status, the meta-analysis was rerun using only the studies which clearly reported that AD patients were drug naïve or had a washout period before PSG examination. The results were unchanged compared to the full sample analysis.

A subgroup analysis revealed that having an adaptation night or not across different studies was a significant source of heterogeneity between AD patients and controls for differences in WASO and SE. Specifically, significantly increased WASO and decreased SE in AD patients compared with controls were only found in studies using an adaptation night (*p* < 0.05).

Another subgroup analysis revealed that the PSG scoring method (AASM vs. R&K) was a significant source of heterogeneity between AD patients and controls for differences in TST, WASO, SE, and percentage of N1, SWS, and REM sleep (*p* < 0.05). Significantly decreased TST, SE, SWS, and REM sleep percentage, and increased N1 percentage and WASO were observed regardless of scoring criteria used. However, the magnitude of changes in these parameters in AD patients compared with controls were greater in studies using AASM criteria compared with those in studies using R&K criteria (*p* < 0.05).

### Associations of PSG measured sleep with cognitive decline in AD patients

**Fig. 2:**
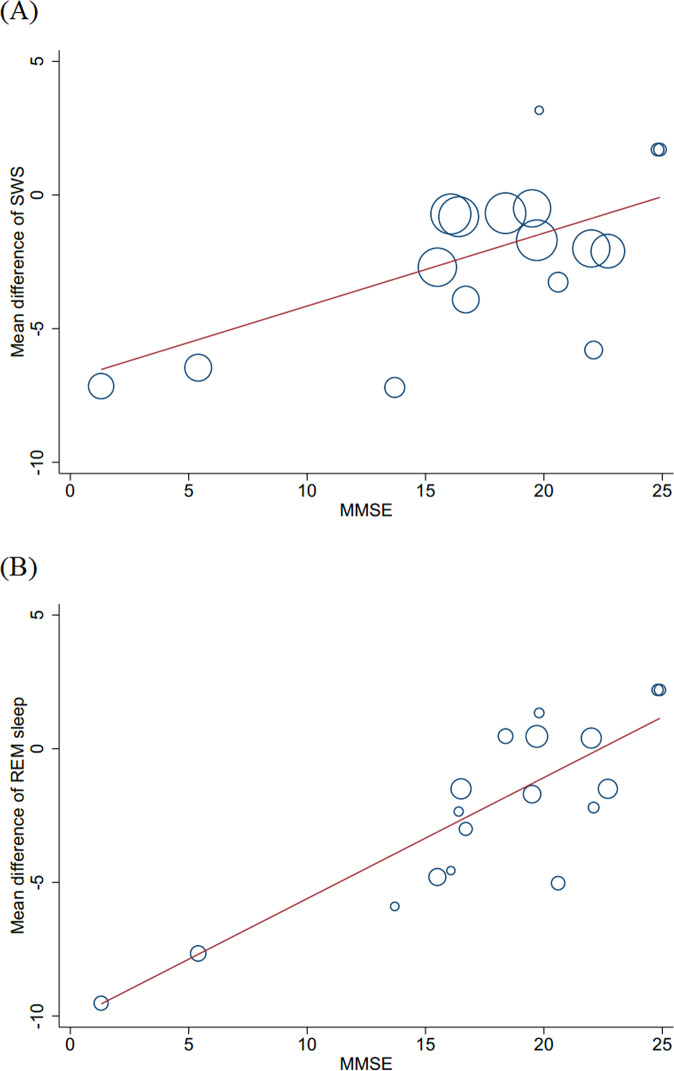
Significant associations between sleep changes and MMSE score in patients with Alzheimer’s disease. **A** Associations of the changes in slow wave sleep (coefficient = 0.273; *p* = 0.004) with MMSE score in patients with Alzheimer’s disease in studies using R&K criteria; **B** Associations of the changes in rapid eye movement sleep (coefficient = 0.453; *p* < 0.001) with MMSE score in patients with Alzheimer’s disease in studies using R&K criteria. MMSE mini-mental state examination.

Meta-regression analysis did not reveal any significant associations between sleep measures and MMSE score in AD patients compared to controls; although associations between decreased SWS and REM sleep percentage and decreased MMSE score in AD patients approached statistical significance, *p* = 0.075 and *p* = 0.071, respectively. Given that PSG scoring rules (AASM vs. R&K) were found to be a significant source of heterogeneity for SWS and REM sleep differences, a second analysis was conducted with studies using R&K criteria, which revealed statistically significant associations between decreased MMSE with decreased SWS (*p* = 0.004) and REM sleep (*p* < 0.001) percentage in AD patients (Fig. 2). A comparable analysis for studies using AASM criteria was not possible because of limited available data.

### Sleep parameters which could not be meta-analyzed

PSA data [41, 42, 48, 49, 52, 59], CAP parameters [46], and sleep spindles [25, 40, 45, 47, 49] were also explored for possible differences between AD patients and controls (Table S8). However meta-analytic evaluation of these parameters was not possible due to the limited number of available studies and methodological differences across studies.

## Discussion

### Summary of findings

To our knowledge, this is the first systematic review and meta-analysis to explore how the PSG changes in AD patients. The results showed decreased TST, SE, SWS and REM sleep percentage, and REMD, and increased number of awakenings, WASO, N1 percentage, SL, and REML in AD patients compared with controls. Importantly, decreased SWS and REM sleep percentage were significantly associated with decreased MMSE scores in AD patients, although these associations were found only in studies using R&K scoring criteria. Sophisticated analyses of sleep microstructure (i.e., PSA, CAP, etc.), while studied in AD, could not be meta-analyzed because of the limited number of studies that have reported on these parameters.

### Sleep changes in AD

Our systematic review showed that sleep continuity and architecture (decreased TST, SWS, and REM sleep) are disturbed in AD patients. Accumulating evidence shows that sleep disturbance contributes to cognitive decline and may increase the risk of AD dementia [8, 10, 60–62]. A meta-analysis of 18 longitudinal studies revealed that sleep disturbances predict the development of AD dementia [10]. Supporting this hypothesis are reports that similar alterations in PSG measured sleep parameters including increased N1 sleep, WASO, SL, REML, and decreased TST, SE, and REM sleep are found in individuals with mild cognitive impairment, a prodromal stage of AD [63]. Taken together, these findings suggest a bidirectional relationship between sleep and AD [8].

Circadian dysrhythmia is commonly observed in AD patients and has been considered to be a major cause of their sleep problems [64, 65]. For instance, a 24-h PSG study in AD patients revealed obvious fragmentation in the sleep and waking rhythm with nighttime wakefulness periods and frequent daytime napping [49]. Circadian dysrhythmia in AD patients is likely the result of progressive neuropathological changes in brain regions that play an important role in circadian regulation, such as the suprachiasmatic nucleus [61].

Furthermore, sleep disturbance has been demonstrated to be associated with increased inflammation activation [66]. Studies have also suggested that inflammation, which may promote Aβ accumulation, might be a biological risk factor for mild cognitive impairment preceding the AD onset [67, 68]. Thus, inflammation is hypothesized to be a biologically plausible pathway linking sleep disturbance and increased risk of AD [69, 70]. It has also been speculated that treating sleep disturbances may mitigate inflammatory processes potentially ameliorating the development of AD (see Irwin and Vitiello [8] for more details on inflammation as an underlying mechanism of the sleep/AD relationship).

### Associations of changes in sleep macrostructure with AD severity

Clinically, it is important to ask which PSG parameter changes are associated with progressive memory loss and decline in global cognition, the core symptoms of AD. Our meta-regression analysis revealed significant associations of decreased SWS and REM sleep with decreased MMSE scores. Previous studies have indicated that SWS benefits the consolidation of declarative memories [71–73]. Disruptions in slow-wave activity increase Aβ levels and are associated with impairments of learning, memory, attention, and executive processes [16, 74]. REM sleep benefits non-declarative (emotional and procedural) memory [71, 72, 75], and loss of REM sleep impacts non-declarative memory consolidation [18, 76, 77]. These findings indicate potential contributions of disturbed SWS and REM sleep to the cognitive impairments seen in AD and suggest that developing sleep therapies improving SWS and REM sleep may have potential for slowing cognitive decline in AD if begun early enough in the disease trajectory. Furthermore, given that MMSE is one of the most widely used tools to reflect the severity of cognitive decline or stage/progression of dementia, decreased SWS and REM sleep are also useful indicators to reflect the severity of cognitive decline or stage/progression of dementia. It should be noted that the associations of SWS and REM sleep with MMSE performance were found only in studies using R&K scoring rules and that studies using AASM scoring rules could not be separately examined because of limited available data. Although the differences between the AASM and R&K scoring rules are minor, SWS and REM sleep in the same participants could vary with the application of different scoring rules [78]. Thus, new studies using AASM scoring rules are needed to confirm our findings.

### Effects of sex, age, and education level on sleep in AD

It has been demonstrated that being female and having advanced age are highly significant risk factors for AD [79]. By comparison, high education level is a protective factor against AD, and previous findings suggest that the risk of dementia may be decreased by 7% for each year of additional education [80]. Our findings revealed that being female and of advanced age were associated with increased SL in AD patients compared with controls and that higher education level was associated with less N1 sleep in AD patients compared with controls. Together, these findings suggest that shorter SL and less N1 sleep might be beneficial for AD patients, again indicating the potential usefulness of strategies to improve sleep that might be beneficial for those at risk for or at an early stage of AD.

The mechanisms underlying the relationships of sex, age, and education to sleep changes in AD are unclear. We speculate that they may impact sleep by moderating AD pathology. Regarding the sex difference, Nebel et al. suggested that the increased risk of AD in women may be attributed to a relative lack of some protective factors, such as estrogen deficiency of post-menopausal women, with increased vulnerability to AD pathology [81]. Regarding age, AD pathology including intracellular neurofibrillary tangles and extracellular senile plaques is worse with advanced age in AD patients [1, 82]. Regarding education, neuroimaging studies revealed that education level is positively associated with brain reserves including regional cortical thickness [83] and white and gray matter volume [84]. In addition, high education level may be associated with protection against developing AD pathology, including tau [85] and Aβ [86].

### Sleep spindle and PSA

Sleep spindles, a key EEG feature of N2 sleep are generated by a complex interaction between thalamic, limbic, and cortical regions [87], and can be compromised by disruptions in the structural and functional integrity of these regions from neurodegenerative diseases [88]. These factors suggest that altered sleep spindles may be a potential biomarker of neurodegenerative disease [88]. Liu et al. reported that AD patients had poorer spindle activity compared with controls, and this alteration was associated with decreased MMSE and Montreal Cognitive Assessment scores [45]. Rauchs et al. reported that sleep spindles were globally reduced in aging and AD, while AD patients also exhibited a further decrease in fast spindles compared with normally aging individuals [25]. Furthermore, the mean intensity of fast spindles was positively associated with immediate recall performance in AD patients [25]. These findings suggest that monitoring sleep spindles could be of clinical and biomarker relevance for diagnosing AD. However, both the sensitivity and specificity of sleep spindles for diagnosing AD need to be fully delineated.

Previous studies suggested that quantifying changes in EEG frequency components could provide important neurobiological insight into the disease and its clinical relevance. For instance, previous studies suggested that EEG slowing during REM sleep, which is related to the degeneration of the cholinergic structures in the brain stem and forebrain [89], may be a biological marker of AD [41, 49] although its sensitivity and specificity for diagnosing AD are also unclear. Additional evidence showed that increases in the faster theta frequency band in SWS is significantly linked to better delayed episodic recall in AD patients, suggesting a potential compensatory mechanism during SWS against the impact of AD pathology [42]. However, it is difficult to make unequivocal conclusions regarding the relevance of EEG frequency components in AD, because of limited available studies and methodological differences across studies.

### Limitations

This review has limitations. First, some factors, such as variations in bedtime schedule across sleep labs and discomfort from wearing PSG devices, which may potentially impact the pooled effect sizes of sleep changes, could not be accounted for in our meta-analysis. Second, some factors, such as daytime sleepiness, depression, and anxiety which also potentially influence PSG changes in AD could not be explored due to limited available data for these variables. Third, it should be noted that obstructive sleep apnea (OSA) may significantly impact sleep [90–92]. In the 24 studies included in our meta-analysis, the majority of studies did not report whether they excluded OSA, while the eight studies that stated that they excluded the OSA used a variety of exclusion criteria. Thus, the potential effects of OSA on our findings are unknown. Fourth, although a majority of our included studies reported that their participants were mild to moderate AD patients, the detailed disease progression/stage (when the PSG examination was performed) was still unclear, which may be associated with the effect sizes of PSG changes in AD. These limitations suggest that our findings should be interpreted with caution.

## Conclusions

The present study reports the first in-depth exploration of the existing literature on PSG measured sleep changes in AD. Although there was methodological heterogeneity across the included studies, this systematic review clearly identified disrupted objective sleep as a significant problem in AD. Importantly, decreased SWS and REM sleep are associated with impairments in cognitive function and are potential targets for therapeutic intervention aimed at improving both sleep and cognitive function in mild cognitive impairment and mild stage AD. Furthermore, alterations in sleep spindles and EEG frequency activity may be clinically useful for diagnosing AD but further studies are needed to confirm this possibility.

## Supplementary information

Supplementary flie
