# Supplementary material for: Sleep in Alzheimer’s disease: a systematic review and meta-analysis of polysomnographic findings
Source: Transl Psychiatry. 2022 Apr 1;12:136. doi: 10.1038/s41398-022-01897-y (PMC8976015; doi:10.1038/s41398-022-01897-y)

**Table S1.** Ovid MEDLINE(R) and Epub Ahead of Print, In-Process, In-Data-Review & Other Non-Indexed Citations, Daily and Versions(R) 1946 to March 05, 2021 (Up to Mar 6, 2021).

| \|  \| **#** \| **Searches** \| **Results** \| \| --- \| --- \| --- \| --- \| \|  \| 1. \| exp Alzheimer Disease/ \| 97430 \| \|  \| 2. \| (Alzheimer Disease or Alzheimer's disease).ab,ti. \| 136856 \| \|  \| 3. \| 1 or 2 \| 155602 \| \|  \| 4. \| exp POLYSOMNOGRAPHY/ \| 21903 \| \|  \| 5. \| (polysomnogra* or PSG or sleep architect* or sleep monit* or sleep stage*).ab,ti. \| 25188 \| \|  \| 6. \| exp Electroencephalography/ \| 166726 \| \|  \| 7. \| (electroencephalogra* or EEG).ab,ti. \| 104932 \| \|  \| 8. \| 4 or 5 or 6 or 7 \| 222229 \| \|  \| 9. \| 3 and 8 \| 2399 \| \|  \| 10. \| exp animal/ not human/ \| 4796559 \| \|  \| 11. \| 9 not 10 \| 2236 \| \|  \| 12. \| limit 11 to english language \| 2038 \| |
| --- | --- | --- | --- | --- | --- | --- | --- | --- | --- | --- | --- | --- | --- | --- | --- | --- | --- | --- | --- | --- | --- | --- | --- | --- | --- | --- | --- | --- | --- | --- | --- | --- | --- | --- | --- | --- | --- | --- | --- | --- | --- | --- | --- | --- | --- | --- | --- | --- | --- | --- | --- | --- |

**Table S2.** Embase 1974 to 2021 March 05 (Up to Mar 6, 2021).

| \| **#** \| **Searches** \| **Results** \| \| --- \| --- \| --- \| \| 1. \| exp Alzheimer disease/ \| 209242 \| \| 2. \| (Alzheimer Disease or Alzheimer's disease).ab,ti. \| 187365 \| \| 3. \| 1 or 2 \| 241206 \| \| 4. \| exp polysomnography/ \| 40594 \| \| 5. \| (polysomnogra* or PSG or sleep architect* or sleep monit* or sleep stage*).ab,ti. \| 45800 \| \| 6. \| exp electroencephalogram/ \| 135023 \| \| 7. \| (electroencephalogra* or EEG).ab,ti. \| 141085 \| \| 8. \| 4 or 5 or 6 or 7 \| 246600 \| \| 9. \| 3 and 8 \| 4371 \| \| 10. \| exp animal/ not human/ \| 4914686 \| \| 11. \| 9 not 10 \| 3973 \| \| 12. \| limit 11 to english language \| 3734 \| |
| --- | --- | --- | --- | --- | --- | --- | --- | --- | --- | --- | --- | --- | --- | --- | --- | --- | --- | --- | --- | --- | --- | --- | --- | --- | --- | --- | --- | --- | --- | --- | --- | --- | --- | --- | --- | --- | --- | --- | --- |

**Table S3.** EBM Reviews - Cochrane Database of Systematic Reviews 2005 to March 3, 2021, Database Field Guide EBM Reviews - ACP Journal Club 1991 to February 2021, Database Field Guide EBM Reviews - Database of Abstracts of Reviews of Effects 1st Quarter 2016, Database Field Guide EBM Reviews - Cochrane Clinical Answers February 2021, Database Field Guide EBM Reviews - Cochrane Central Register of Controlled Trials February 2021, Database Field Guide EBM Reviews - Cochrane Methodology Register 3rd Quarter 2012, Database Field Guide EBM Reviews - Health Technology Assessment 4th Quarter 2016, Database Field Guide EBM Reviews - NHS Economic Evaluation Database 1st Quarter 2016 (Up to Mar 6, 2021).

| \| **#** \| **Searches** \|  \| **Results** \| \| --- \| --- \| --- \| --- \| \| 1. \| (Alzheimer Disease or Alzheimer's disease).ab,ti. \|  \| 10630 \| \| 2. \| (polysomnogra* or PSG or sleep architect* or sleep monit* or sleep stage*).ab,ti. \|  \| 5252 \| \| 3. \| (electroencephalogra* or EEG).ab,ti. \|  \| 9294 \| \| 4. \| 2 or 3 \|  \| 14029 \| \| 5. \| 1 and 4 \|  \| 279 \| |
| --- | --- | --- | --- | --- | --- | --- | --- | --- | --- | --- | --- | --- | --- | --- | --- | --- | --- | --- | --- | --- | --- | --- | --- | --- |

**Table S4.** PsycINFO via EBSCO*host* (Up to Mar 6, 2021).

| # | Searches | **Actions** | Results |
| --- | --- | --- | --- |
| S12 | S10 AND S11 | **Limiters** - English; Population Group: Human  **Search modes** - Boolean/Phrase | 1,223 |
| S11 | S4 OR S5 OR S6 OR S7 OR S8 OR S9 | **Search modes** - Boolean/Phrase | 76,834 |
| S10 | S1 OR S2 OR S3 | **Search modes** - Boolean/Phrase | 63,649 |
| S9 | DE "Polysomnography" | **Search modes** - Boolean/Phrase | 5,489 |
| S8 | TI electroencephalogra* OR AB electroencephalogra* OR TI EEG OR AB EEG | **Search modes** - Boolean/Phrase | 45,089 |
| S7 | TI polysomnogra* OR AB polysomnogra* OR TI PSG OR AB PSG OR TI sleep architect* OR AB sleep architect* OR TI sleep monit* OR AB sleep monit* OR TI sleep stage* OR AB sleep stage* | **Search modes** - Boolean/Phrase | 9,754 |
| S6 | MA Electroencephalography | **Search modes** - Boolean/Phrase | 37,740 |
| S5 | DE "Electroencephalography" OR DE "Alpha Rhythm" OR DE "Beta Rhythm" OR DE "Delta Rhythm" OR DE "Gamma Rhythm" OR DE "Theta Rhythm" | **Search modes** - Boolean/Phrase | 56,360 |
| S4 | MA POLYSOMNOGRAPHY | **Search modes** - Boolean/Phrase | 4,618 |
| S3 | TI Alzheimer Disease OR AB Alzheimer Disease OR TI Alzheimer's disease OR AB Alzheimer's disease | **Search modes** - Boolean/Phrase | 56,527 |
| S2 | MA Alzheimer Disease | **Search modes** - Boolean/Phrase | 28,318 |
| S1 | DE "Alzheimer's Disease" | **Search modes** - Boolean/Phrase | 48,306 |

**Table S5.** CINAHL plus with full text via EBSCO*host* (Up to Mar 6, 2021).

| **#** | **Searches** | **Actions** | **Results** |
| --- | --- | --- | --- |
| S8 | S6 AND S7 | **Limiters** - Human; Language: English  **Search modes** - Boolean/Phrase | 259 |
| S7 | S3 OR S4 OR S5 | **Search modes** - Boolean/Phrase | 31,771 |
| S6 | S1 OR S2 | **Search modes** - Boolean/Phrase | 42,764 |
| S5 | TI electroencephalogra* OR AB electroencephalogra* OR TI EEG OR AB EEG | **Search modes** - Boolean/Phrase | 11,971 |
| S4 | TI polysomnogra* OR AB polysomnogra* OR TI PSG OR AB PSG OR TI sleep architect* OR AB sleep architect* OR TI sleep monit* OR AB sleep monit* OR TI sleep stage* OR AB sleep stage* | **Search modes** - Boolean/Phrase | 7,256 |
| S3 | (MH "Polysomnography") OR (MH "Electroencephalography") | **Search modes** - Boolean/Phrase | 25,352 |
| S2 | TI Alzheimer Disease OR AB Alzheimer Disease OR TI Alzheimer's disease OR AB Alzheimer's disease | **Search modes** - Boolean/Phrase | 30,800 |
| S1 | (MH "Alzheimer's Disease") | **Search modes** - Boolean/Phrase | 33,358 |

**Table S6.** Studies excluded with reasons

| **Studies** | **Reasons** |
| --- | --- |
| Bliwise et al., 2012 [1] | Not the target controls |
| Rauchs et al., 2013 [2] | Overlapping data |
| Zhang et al., 2019 [3] | Not the target patients |
| Yin et al., 2015 [4] | Not the target controls |
| Yesavage et al., 2002 [5] | No target outcome |
| Yesavage et al., 2004 [6] | Not the target controls |
| Yamadera et al., 2000 [7] | Not the target controls |
| Wunderlin et al., 2020 [8] | Review paper |
| Winer et al., 2020 [9] | Not the target controls |
| Wirsich et al., 2018 [10] | Not the target patients |
| Winer et al., 2019 [11] | Not the target controls |
| Weissova et al., 2016 [12] | No target outcome |
| Wang et al., 2020 [13] | Not the target patients |
| Wang et al., 2016 [14] | Not the target controls |
| von Känel et al., 2006 [15] | Not the target patients |
| Vitiello et al., 2002 [16] | No target outcome |
| Varga et al., 2016 [17] | Not the target patients |
| Troussière et al., 2014 [18] | Not the target controls |
| Thomas et al., 2020 [19] | Not the target patients |
| Thomas et al.,, 2019 [20] | Not the target patients |
| Targa et al., 2021 [21] | Not the target controls |
| Taillard et al., [22] | Not the target patients |
| Tadokoro et al., 2020 [23] | No target outcome |
| Staner, 2002 [24] | Review paper |
| Spira et al., 2014 [25] | Not the target patients |
| Singer te al., 2003 [26] | Not the target controls |
| Schredl et al., 2005 [27] | Not the target controls |
| Satlin et al., 1992 [28] | Not the target controls |
| Rowe et al., 2008 [29] | Not the target patients |
| Reynolds et al., 1985 [30] | Overlapping data |
| Reynolds et al., 1990 [31] | Overlapping data |
| Przybylska-Kuc et al., 2019 [32] | Not the target patients |
| Petit et al., 1993 [33] | Not the target controls |
| Petit et al., 1993 [34] | Overlapping data |
| Pase et al., 2017 [35] | Not the study design |
| Park et al., 2012 [36] | Not the target controls |
| Ooms et al., 2014 [37] | Not the target patients |
| Okawa et al., 1991 [38] | Not the target patients |
| Muto et al., 2021 [39] | Not the target patients |
| Mulin et al., 2011 [40] | Not the target controls |
| Most et al., 2012 [41] | No target outcome |
| Most et al., 2012 [42] | No target outcome |
| Montplaisir et al.,1998 [43] | No target outcome |
| Mizuno et al., 2004 [44] | Not the target controls |
| Mishima et al., 1999 [45] | No target outcome |
| Mills et al., 2009 [46] | Not the target patients |
| Meghdadi et al., 2019 [47] | No target outcome |
| McCurry et al., 2006 [48] | No target outcome |
| McCurry et al., 2008 [49] | No target outcome |
| McCarter et al., 2020 [50] | No target outcome |
| Manousakis et al., 2018 [51] | Not the target patients |
| Manni et al., 2013 [52] | Not the target controls |
| Manni et al., 2015 [53] | Not the target controls |
| Mamelak, 1997 [54] | Review paper |
| Maia et al., 2019 [55] | No target outcome |
| Lutsey et al., 2016 [56] | No target outcome |
| Lucey et al., 2019 [57] | No target outcome |
| Loewenstein et al., 1982 [58] | Overlapping data |
| Liu et al., 2020 [59] | Not the target patients |
| Lim et al., 2014 [60] | No target outcome |
| Liguori et al., 2014 [61] | Overlapping data |
| Liguori et al., 2016 [62] | Not the target patients |
| Liguori et al., 2018 [63] | Not the target controls |
| Liguori et al., 2017 [64] | Overlapping data |
| Liguori et al., 2017 [65] | Not the target patients |
| Ladenbauer et al., 2017 [66] | No target outcome |
| Kundermann et al., 2011 [67] | Not the target controls |
| Kheirandish-Gozal et al., 2019 [68] | Review paper |
| Karlinsky et al., 1992 [69] | No target outcome |
| Kanbayashi et al., 2002 [70] | Not the target controls |
| Kam et al., 2019 [71] | Not the target patients |
| Kabeshita et al., 2017 [72] | Not the target controls |
| Ju et al., 2017 [73] | Not the target controls |
| Ju et al., 2019 [74] | Not the target patients |
| Ju et al., 2016 [75] | Not the target patients |
| Jorge et al., 2020 [76] | Not the target controls |
| Johnson et al., 2017 [77] | Not the study design |
| Ismail et al., 2009 [78] | No target outcome |
| Ishikawa et al., 2016 [79] | Not the target controls |
| Honda et al., 2013 [80] | No target outcome |
| Hoch et al., 1988 [81] | Overlapping data |
| Hita-Yanez et al., 2013 [82] | Not the target patients |
| Higami et al., 2019 [83] | Not the target controls |
| Hibi et al., 2012 [84] | Not the target controls |
| Herring et al., 2020 [85] | Not the target controls |
| Helkala et al., 1996 [86] | No target outcome |
| Helkala et al., 1991 [87] | No target outcome |
| Hanin et al., 1984 [88] | No target outcome |
| Handa et al., 2019 [89] | Not the target controls |
| Grossberg, 2017 [90] | Review paper |
| Grace et al., 2000 [91] | Not the target controls |
| Gabelle et al., 2017 [92] | Not the target patients |
| Foster et al., 1987 [93] | Not the target controls |
| Fernandez et al., 2002 [94] | No target outcome |
| Ferman et al., 2014 [95] | Not the target controls |
| Ferman et al., 2002 [96] | Not the target controls |
| Ferman et al., 1999 [97] | Not the target controls |
| Diaz-Roma et al., 2021 [98] | Not the target patients |
| Dede et al., 2019 [99] | Not the target patients |
| Cooke et al., 2009 [100] | Not the target patients |
| Cooke et al., 2006 [101] | Not the target controls |
| Cooke et al., 2006 [102] | Not the target controls |
| Cook et al., 2020 [103] | Review paper |
| Cole et al., 2010 [104] | Not the target controls |
| Chong et al., 2006 [105] | Not the target controls |
| Chen et al., 2011 [106] | No target outcome |
| Carnicelli et al., 2019 [107] | Not the target patients |
| Boeve et al., 2001 [108] | Not the target controls |
| Bliwise et al., 1995 [109] | Not the target controls |
| Bliwise et al., 2012 [110] | Not the target controls |
| Bliwise et al., 1995 [111] | Not the target controls |
| Baumann et al., 2004 [112] | No target outcome |
| Andre et al., 2020 [113] | Not the target patients |
| Allen et al., 1987 [114] | Not the target controls |
| Moe et al., 1993 [115] | Overlapping data |

**References of excluded studies**

[1] Bliwise DL, Trotti LM, Yesavage JA, Rye DB. Periodic leg movements in sleep in elderly patients with Parkinsonism and Alzheimer's disease. European journal of neurology. 2012;19:918-23.

[2] Rauchs G, Piolino P, Bertran F, de La Sayette V, Viader F, Eustache F, et al. Retrieval of recent autobiographical memories is associated with slow-wave sleep in early AD. Frontiers in behavioral neuroscience. 2013;7:114.

[3] Zhang F, Zhong R, Li S, Fu Z, Wang R, Wang T, et al. Alteration in sleep architecture and electroencephalogram as an early sign of Alzheimer's disease preceding the disease pathology and cognitive decline. Alzheimer's & Dementia: The Journal of the Alzheimer's Association. 2019;15:590-7.

[4] Yin Y, Liu Y, Zhuang J, Pan X, Li P, Yang Y, et al. Low-dose atypical antipsychotic risperidone improves the 5-Year outcome in Alzheimer's disease patients with sleep disturbances. Pharmacology. 2015;96:155.

[5] Yesavage JA, Taylor JL, Kraemer H, Noda A, Friedman L, Tinklenberg JR, et al. Sleep/wake cycle disturbance in Alzheimer's disease: how much is due to an inherent trait? International Psychogeriatrics. 2002:73-81.

[6] Yesavage JA, Friedman L, Kraemer H, Tinklenberg JR, Salehi A, Noda A, et al. Sleep/wake disruption in Alzheimer's disease: APOE status and longitudinal course. Journal of geriatric psychiatry and neurology. 2004;17:20-4.

[7] Yamadera H, Ito T, Suzuki H, Asayama K, Ito R, Endo S. Effects of bright light on cognitive and sleep-wake (circadian) rhythm disturbances in Alzheimer-type dementia. Psychiatry and clinical neurosciences. 2000;54:352-3.

[8] Wunderlin M, Züst MA, Fehér KD, Klöppel S, Nissen C. The role of slow wave sleep in the development of dementia and its potential for preventative interventions. Psychiatry Research: Neuroimaging. 2020;306.

[9] Winer JR, Mander BA, Kumar S, Reed M, Baker SL, Jagust WJ, et al. Sleep disturbance forecasts beta-amyloid accumulation across subsequent years. Current biology : CB. 2020;30:4291-8.

[10] Wirsich J, Rey M, Guye M, Benar C, Lanteaume L, Ridley B, et al. Brain networks are independently modulated by donepezil, sleep, and sleep deprivation. Brain topography. 2018;31:380-91.

[11] Winer JR, Mander BA, Helfrich RF, Maass A, Harrison TM, Baker SL, et al. Sleep as a potential biomarker of tau and beta-amyloid burden in the human brain. The Journal of neuroscience. 2019;39:6315-24.

[12] Weissova K, Bartos A, Sladek M, Novakova M, Sumova A. Moderate changes in the circadian system of Alzheimer's disease patients detected in their home environment. PloS one. 2016;11:e0146200.

[13] Wang Y, Cheng C, Moelter S, Fuentecilla JL, Kincheloe K, Lozano AJ, et al. One Year of Continuous positive airway pressure adherence improves cognition in older adults with mild apnea and mild cognitive impairment. Nursing research. 2020;69:157-64.

[14] Wang P, Wing YK, Xing J, Liu Y, Zhou B, Zhang Z, et al. Rapid eye movement sleep behavior disorder in patients with probable Alzheimer's disease. Aging clinical and experimental research. 2016;28:951-7.

[15] von Känel R, Dimsdale JE, Ancoli-Israel S, Mills PJ, Patterson TL, McKibbin CL, et al. Poor sleep is associated with higher plasma proinflammatory cytokine interleukin-6 and procoagulant marker fibrin d-dimer in older caregivers of people with Alzheimer's disease. Journal of the American Geriatrics Society. 2006;54:431-7.

[16] Vitiello MV, Moe KE, Prinz PN. Sleep complaints cosegregate with illness in older adults clinical research informed by and informing epidemiological studies of sleep. Journal of psychosomatic research. 2002;53:555-9.

[17] Varga AW, Wohlleber ME, Gimenez S, Romero S, Alonso JF, Ducca EL, et al. Reduced slow-wave sleep is associated with high cerebrospinal fluid abeta42 levels in cognitively normal elderly. Sleep. 2016;39:2041-8.

[18] Troussière A-C, Charley CM, Salleron J, Richard F, Delbeuck X, Derambure P, et al. Treatment of sleep apnoea syndrome decreases cognitive decline in patients with Alzheimer's disease. Journal of Neurology, Neurosurgery & Psychiatry. 2014;85:1405-8.

[19] Thomas J, Ooms SJ, Mentink LJ, Booij J, Olde Rikkert MGM, Overeem S, et al. Effects of long-term sleep disruption on cognitive function and brain amyloid-beta burden: A case-control study. Alzheimer's Research and Therapy. 2020;12:101.

[20] Thomas J, Ooms S, Verbeek M, Booij J, Rijpkema M, Kessels RPC, et al. Sleep-cognition hypothesis in maritime pilots, what is the effect of long-term work-related poor sleep on cognition and amyloid accumulation in healthy middle-aged maritime pilots: Methodology of a case-control study. BMJ open. 2019;9:e026992.

[21] Targa A, Dakterzada F, Benitez I, Lopez R, Pujol M, Dalmases M, et al. Decrease in sleep depth is associated with higher cerebrospinal fluid neurofilament light levels in patients with Alzheimer's disease. Sleep. 2021;44.

[22] Taillard J, Sagaspe P, Berthomier C, Brandewinder M, Amieva H, Dartigues J-F, et al. Non-REM sleep characteristics predict early cognitive impairment in an aging population. Frontiers in neurology. 2019;10:197.

[23] Tadokoro K, Ohta Y, Hishikawa N, Nomura E, Wakutani Y, Takao Y, et al. Discrepancy of subjective and objective sleep problems in Alzheimer's disease and mild cognitive impairment detected by a home-based sleep analysis. Journal of clinical neuroscience : official journal of the Neurosurgical Society of Australasia. 2020;74:76-80.

[24] Staner L. Sleep-wake mechanisms and drug discovery: Sleep EEG as a tool for the development of CNS-acting drugs. Dialogues in clinical neuroscience. 2002;4:342-50.

[25] Spira AP, Yager C, Brandt J, Smith GS, Zhou Y, Mathur A, et al. Objectively measured sleep and beta-amyloid burden in older adults: A Pilot Study. SAGE open medicine. 2014;2.

[26] Singer C, Tractenberg RE, Kaye J, Schafer K, Gamst A, Grundman M, et al. A multicenter, placebo-controlled trial of melatonin for sleep disturbance in Alzheimer's disease. Sleep. 2003;26:893-901.

[27] Schredl M, Speck S. The effect of donepezil on sleep in patients with Alzheimer's disease: an open pilot study. Sleep & Hypnosis. 2005;7:63-7.

[28] Satlin A, Volicer L, Ross V, Herz LR, Campbell S. Bright light treatment of behavioral and sleep disturbances in patients with Alzheimer's disease. Am J Psychiatry. 1992;149:1028-32.

[29] Rowe MA, McCrae CS, Campbell JM, Benito AP, Cheng J. Sleep pattern differences between older adult dementia caregivers and older adult noncaregivers using objective and subjective measures. Journal of Clinical Sleep Medicine. 2008;4:362-9.

[30] Reynolds CF, Kupfer DJ, Taska LS, Hoch CC, Sewitch DE, Restifo K, et al. Sleep apnea in Alzheimer's dementia: Correlation with mental deterioration. The Journal of clinical psychiatry. 1985;46:257-61.

[31] Reynolds CF, Buysse DJ, Kupfer DJ, Hoch CC, Houck PR, Matzzie J, et al. Rapid eye movement sleep deprivation as a probe in elderly subjects. Archives of general psychiatry. 1990;47:1128-36.

[32] Przybylska-Kuc S, Zakrzewski M, Dybala A, Kicinski P, Dzida G, Myslinski W, et al. Obstructive sleep apnea may increase the risk of Alzheimer's disease. PloS one. 2019;14:e0221255.

[33] Petit D, Montplaisir J, Lorrain D, Gauthier S. THA does not affect sleep or EEG spectral power in Alzheimer's disease. Biological psychiatry. 1993;33:753-4.

[34] Petit D, Lorrain D, Gauthier S, Montplaisir J. Regional spectral analysis of the REM sleep EEG in mild to moderate Alzheimer's disease. Neurobiology of aging. 1993;14:141-5.

[35] Pase MP, Himali JJ, Grima NA, Beiser AS, Satizabal CL, Aparicio HJ, et al. Sleep architecture and the risk of incident dementia in the community. Neurology. 2017;89:1244-50.

[36] Park M, Hood MM, Shah RC, Fogg LF, Wyatt JK. Sleepiness, parkinsonian features and sustained attention in mild Alzheimer's disease. Age and ageing. 2012;41:765-70.

[37] Ooms S, Overeem S, Besse K, Rikkert MO, Verbeek M, Claassen JAHR. Effect of 1 Night of total sleep deprivation on cerebrospinal fluid β-amyloid 42 in healthy middle-aged men: A randomized clinical trial. JAMA neurology. 2014;71:971-7.

[38] Okawa M, Mishima K, Hishikawa Y, Hozumi S, Hori H, Takahashi K. Circadian rhythm disorders in sleep-waking and body temperature in elderly patients with dementia and their treatment. Sleep. 1991;14:478-85.

[39] Muto V, Koshmanova E, Ghaemmaghami P, Jaspar M, Meyer C, Elansary M, et al. Alzheimer's disease genetic risk and sleep phenotypes in healthy young men: Association with more slow waves and daytime sleepiness. Sleep. 2021;44:zsaa137.

[40] Mulin E, Zeitzer JM, Friedman L, Duff FL, Yesavage J, Robert PH, et al. Relationship between apathy and sleep disturbance in mild and moderate Alzheimer’s disease: An actigraphic study. Journal of Alzheimer's Disease. 2011;25:85-91.

[41] Most EIS, Scheltens P, Van Someren EJW. Increased skin temperature in Alzheimer’s disease is associated with sleepiness. Journal of Neural Transmission. 2012;119:1185-94.

[42] Most EIS, Aboudan S, Scheltens P, Van Someren EJW. Discrepancy between subjective and objective sleep disturbances in early- and moderate-stage Alzheimer disease. The American Journal of Geriatric Psychiatry. 2012;20:460-7.

[43] Montplaisir J, Petit D, Gauthier S, Gaudreau H, Decary A. Sleep disturbances and eeg slowing in alzheimer's disease. Sleep research online : SRO. 1998;1:147-51.

[44] Mizuno S, Kameda A, Inagaki T, Horiguchi J. Effects of donepezil on Alzheimer's disease: The relationship between cognitive function and rapid eye movement sleep. Psychiatry and clinical neurosciences. 2004;58:660-5.

[45] Mishima K, Tozawa T, Satoh K, Matsumoto Y, Hishikawa Y, Okawa M. Melatonin secretion rhythm disorders in patients with senile dementia of Alzheimer's type with disturbed sleep–waking. Biological psychiatry. 1999;45:417-21.

[46] Mills PJ, Ancoli-Israel S, von Känel R, Mausbach BT, Aschbacher K, Patterson TL, et al. Effects of gender and dementia severity on Alzheimer's disease caregivers' sleep and biomarkers of coagulation and inflammation. Brain, behavior, and immunity. 2009;23:605-10.

[47] Meghdadi AH, Popovic D, Rupp G, Smith S, Berka C, Verma A. Transcranial impedance changes during sleep: A rheoencephalography study. IEEE Journal of Translational Engineering in Health and Medicine. 2019;7:8638835.

[48] McCurry SM, Vitiello MV, Gibbons LE, Logsdon RG, Teri L. Factors associated with caregiver reports of sleep disturbances in persons with dementia. The American Journal of Geriatric Psychiatry. 2006;14:112-20.

[49] McCurry SM, Pike KC, Vitiello MV, Logsdon RG, Teri L. Factors associated with concordance and variability of sleep quality in persons with Alzheimer's disease and their caregivers. Sleep. 2008;31:741-8.

[50] McCarter SJ, Tabatabai GM, Jong H-Y, Sandness DJ, Timm PC, Johnson KL, et al. REM sleep atonia loss distinguishes synucleinopathy in older adults with cognitive impairment. Neurology. 2020;94:e15-e29.

[51] Manousakis JE, Scovelle AJ, Rajaratnam SMW, Naismith SL, Anderson C. Advanced circadian timing and sleep fragmentation differentially impact on memory complaint subtype in subjective cognitive decline. Journal of Alzheimer's Disease. 2018;66:565-77.

[52] Manni R, Sinforiani E, Zucchella C, Terzaghi M, Rezzani C. A sleep continuity scale in Alzheimer's disease: validation and relationship with cognitive and functional deterioration. Neurological Sciences. 2013;34:701-5.

[53] Manni R, Sinforiani E, Terzaghi M, Rezzani C, Zucchella C. Sleep continuity scale in Alzheimer’s disease (SCADS): Application in daily clinical practice in an Italian center for dementia. Neurological Sciences. 2015;36:469-71.

[54] Mamelak M. Neurodegeneration, sleep, and cerebral energy metabolism: A testable hypothesis. Journal of geriatric psychiatry and neurology. 1997;10:29-32.

[55] Maia PD, Raj A, Kutz JN. Slow-gamma frequencies are optimally guarded against effects of neurodegenerative diseases and traumatic brain injuries. Journal of Computational Neuroscience. 2019;47.

[56] Lutsey PL, Norby FL, Gottesman RF, Mosley T, MacLehose RF, Punjabi NM, et al. Sleep apnea, sleep duration and brain mri markers of cerebral vascular disease and Alzheimer's disease: The atherosclerosis risk in communities study (ARIC). PloS one. 2016;11:e0158758.

[57] Lucey BP, McCullough A, Landsness EC, Toedebusch CD, McLeland JS, Zaza AM, et al. Reduced non-rapid eye movement sleep is associated with tau pathology in early Alzheimer's disease. Science translational medicine. 2019;11:eaau6550.

[58] Loewenstein RJ, Weingartner H, Gillin JC, Kaye W, Ebert M, Mendelson WB. Disturbances of sleep and cognitive functioning in patients with dementia. Neurobiology of aging. 1982;3:371-7.

[59] Liu S, Pan J, Lei Q, He L, Zhong B, Meng Y, et al. Spontaneous K-Complexes may be biomarkers of the progression of amnestic mild cognitive impairment. Sleep medicine. 2020;67:99-109.

[60] Lim ASP, Ellison BA, Wang JL, Yu L, Schneider JA, Buchman AS, et al. Sleep is related to neuron numbers in the ventrolateral preoptic/intermediate nucleus in older adults with and without Alzheimer’s disease. Brain: A Journal of Neurology. 2014;137:2847-61.

[61] Liguori C, Romigi A, Nuccetelli M, Zannino S, Sancesario G, Martorana A, et al. Orexinergic system dysregulation, sleep impairment, and cognitive decline in Alzheimer disease. JAMA neurology. 2014;71:1498-505.

[62] Liguori C, Nuccetelli M, Izzi F, Sancesario G, Romigi A, Martorana A, et al. Rapid eye movement sleep disruption and sleep fragmentation are associated with increased orexin-a cerebrospinal-fluid levels in mild cognitive impairment due to Alzheimer's disease. Neurobiology of aging. 2016;40:120-6.

[63] Liguori C, Mercuri NB, Nuccetelli M, Izzi F, Bernardini S, Placidi F. Cerebrospinal fluid orexin levels and nocturnal sleep disruption in Alzheimer’s disease patients showing neuropsychiatric symptoms. Journal of Alzheimer's Disease. 2018;66:993-9.

[64] Liguori C, Chiaravalloti A, Nuccetelli M, Izzi F, Sancesario G, Cimini A, et al. Hypothalamic dysfunction is related to sleep impairment and CSF biomarkers in Alzheimer's disease. Journal of neurology. 2017;264:2215-23.

[65] Liguori C, Mercuri NB, Izzi F, Romigi A, Cordella A, Sancesario G, et al. Obstructive sleep apnea is associated with early but possibly modifiable Alzheimer’s disease biomarkers changes. Sleep: Journal of Sleep and Sleep Disorders Research. 2017;40:1-10.

[66] Ladenbauer J, Kulzowa N, De Boor R, Avramova E, Grittner U, Floel A. Promoting sleep oscillations and their functional coupling by transcranial stimulation enhances memory consolidation in mild cognitive impairment. Journal of Neuroscience. 2017;37:7111-24.

[67] Kundermann B, Thum A, Rocamora R, Haag A, Krieg J-C, Hemmeter U. Comparison of polysomnographic variables and their relationship to cognitive impairment in patients with Alzheimer’s disease and frontotemporal dementia. Journal of psychiatric research. 2011;45:1585-92.

[68] Kheirandish-Gozal L, Gozal D. Obstructive sleep apnea and inflammation: Proof of concept based on two illustrative cytokines. International journal of molecular sciences. 2019;20:459.

[69] Karlinsky H, Berg JM, Lennox A, Ray PN, George-Hyslop PS, Farrer LA, et al. Monozygotic twins concordant for late-onset probable Alzheimer disease with suspected Alzheimer disease in four sibs. American journal of medical genetics. 1992;44:591-7.

[70] Kanbayashi T, Sugiyama T, Aizawa R, Saito Y, Ogawa Y, Kitajima T, et al. Effects of donepezil (Aricept) on the rapid eye movement sleep of normal subjects. Psychiatry and clinical neurosciences. 2002;56:307-8.

[71] Kam K, Parekh A, Sharma RA, Andrade A, Lewin M, Castillo B, et al. Sleep oscillation-specific associations with Alzheimer’s disease CSF biomarkers: Novel roles for sleep spindles and tau. Molecular Neurodegeneration. 2019;14.

[72] Kabeshita Y, Adachi H, Matsushita M, Kanemoto H, Sato S, Suzuki Y, et al. Sleep disturbances are key symptoms of very early stage Alzheimer disease with behavioral and psychological symptoms: a Japan multi-center cross-sectional study (J-BIRD). International journal of geriatric psychiatry. 2017;32:222-30.

[73] Ju Y-ES, Ooms SJ, Sutphen C, Macauley SL, Zangrilli MA, Jerome G, et al. Slow wave sleep disruption increases cerebrospinal fluid amyloid-β levels. Brain: A Journal of Neurology. 2017;140:2104-11.

[74] Ju YES, Zangrilli MA, Finn MB, Fagan AM, Holtzman DM. Obstructive sleep apnea treatment, slow wave activity, and amyloid-beta. Annals of neurology. 2019;85:291-5.

[75] Ju YES, Finn MB, Sutphen CL, Herries EM, Jerome GM, Ladenson JH, et al. Obstructive sleep apnea decreases central nervous system–derived proteins in the cerebrospinal fluid. Annals of neurology. 2016;80:154-9.

[76] Jorge C, Targa A, Benitez ID, Dakterzada F, Torres G, Minguez O, et al. Obstructive sleep apnoea and cognitive decline in mild-to-moderate Alzheimer's disease. European Respiratory Journal. 2020;56:2000523.

[77] Johnson DA, Lane J, Wang R, Reid M, Djonlagic I, Fitzpatrick AL, et al. Greater cognitive deficits with sleep-disordered breathing among individuals with genetic susceptibility to alzheimer disease: The multi-ethnic study of atherosclerosis. Annals of the American Thoracic Society. 2017;14:1697-705.

[78] Ismail Z, Herrmann N, Francis PL, Rothenburg LS, Lobaugh NJ, Leibovitch FS, et al. A SPECT study of sleep disturbances and Alzheimer's disease. Dementia and geriatric cognitive disorders. 2009;27:254-9.

[79] Ishikawa I, Shinno H, Ando N, Mori T, Nakamura Y. The effect of memantine on sleep architecture and psychiatric symptoms in patients with Alzheimer’s disease. Acta Neuropsychiatrica. 2016;28:157-64.

[80] Honda K, Hashimoto M, Yatabe Y, Kaneda K, Yuki S, Ogawa Y, et al. The usefulness of monitoring sleep talking for the diagnosis of Dementia with Lewy bodies. International Psychogeriatrics. 2013;25:851-8.

[81] Hoch CC, Reynolds CF, III, Houck PR. Sleep patterns in Alzheimer, depressed, and healthy elderly. Western journal of nursing research. 1988;10:239-51.

[82] Hita-Yanez E, Atienza M, Cantero JL. Polysomnographic and subjective sleep markers of mild cognitive impairment. Sleep. 2013;36:1327-34.

[83] Higami Y, Yamakawa M, Shigenobu K, Kamide K, Makimoto K. High frequency of getting out of bed in patients with Alzheimer's disease monitored by non-wearable actigraphy. Geriatrics and Gerontology International. 2019;19:130-4.

[84] Hibi S, Yamaguchi Y, Umeda-Kameyama Y, Yamamoto H, Iijima K, Momose T, et al. The high frequency of periodic limb movements in patients with Lewy body dementia. Journal of psychiatric research. 2012;46:1590-4.

[85] Herring WJ, Ceesay P, Snyder E, Bliwise D, Budd K, Hutzelmann J, et al. Polysomnographic assessment of suvorexant in patients with probable Alzheimer's disease dementia and insomnia: a randomized trial. Alzheimer's and Dementia. 2020;16:541-51.

[86] Helkala EL, Hanninen T, Kononen M, Hartikainen P, Partanen J, Partanen K, et al. Slow-wave activity in the spectral analysis of the electroencephalogram and volumes of hippocampus in subgroups of Alzheimer's disease patients. Behavioral neuroscience. 1996;110:1235-43.

[87] Helkala E-L, Laulumaa V, Soikkeli R, Partanen J, Soininen H, Riekkinen PJ. Slow-wave activity in the spectral analysis of the electroencephalogram is associated with cortical dysfunctions in patients with Alzheimer's disease. Behavioral neuroscience. 1991;105:409-15.

[88] Hanin I, Reynolds ICF, Dupfer DJ. Elevated red blood cell/plasma choline ratio in dementia of the Alzheimer type: Clinical and polysomnographic correlates. Psychiatry research. 1984;13:167-73.

[89] Handa SS, Baba S, Yamashita K, Nishizaka M, Ando S. The severity of obstructive sleep apnea syndrome cannot predict the accumulation of brain amyloid by imaging with [11C]-Pittsburgh compound B PET computed tomography in patients with a normal cognitive function. Annals of Nuclear Medicine. 2019;33:541-4.

[90] Grossberg S. Acetylcholine neuromodulation in normal and abnormal learning and memory: Vigilance control in waking, sleep, autism, amnesia and Alzheimer's disease. Frontiers in neural circuits. 2017;11:82.

[91] Grace JB, Walker MP, McKeith IG. A comparison of sleep profiles in patients with dementia with Lewy bodies and Alzheimer's disease. International journal of geriatric psychiatry. 2000;15:1028-33.

[92] Gabelle A, Gutierrez L-A, Jaussent I, Navucet S, Grasselli C, Bennys K, et al. Excessive sleepiness and longer nighttime in bed increase the risk of cognitive decline in frail elderly subjects: The MAPT-sleep study. Frontiers in aging neuroscience. 2017;9.

[93] Foster NL, VanDerSpek AFL, Aldrich MS, Berent S, Hichwa RH, Sackellares JC, et al. The effect of diazepam sedation on cerebral glucose metabolism in Alzheimer's disease as measured using positron emission tomography. Journal of Cerebral Blood Flow and Metabolism. 1987;7:415-20.

[94] Fernandez A, Maestu F, Amo C, Gil P, Fehr T, Wienbruch C, et al. Focal temporoparietal slow activity in Alzheimer's disease revealed by magnetoencephalography. Biological psychiatry. 2002;52:764-70.

[95] Ferman TJ, Smith GE, Dickson DW, Graff-Radford NR, Lin S-C, Wszolek Z, et al. Abnormal daytime sleepiness in dementia with Lewy bodies compared to Alzheimer's disease using the Multiple Sleep Latency Test. Alzheimer's research & therapy. 2014;6:76.

[96] Ferman TJ, Boeve BF, Smith GE, Silber MH, Lucas JA, Graff-Radford NR, et al. Dementia with Lewy bodies may present as dementia and REM sleep behavior disorder without parkinsonism or hallucinations. Journal of the International Neuropsychological Society. 2002;8:907-14.

[97] Ferman TJ, Boeve BF, Smith GE, Silber MH, Kokmen E, Petersen RC, et al. REM sleep behavior disorder and dementia: cognitive differences when compared with AD. Neurology. 1999;52:951-7.

[98] Diaz-Roman M, Pulopulos MM, Baquero M, Salvador A, Cuevas A, Ferrer I, et al. Obstructive sleep apnea and Alzheimer's disease-related cerebrospinal fluid biomarkers in mild cognitive impairment. Sleep. 2021;44:zsaa133.

[99] Dede HO, Benbir Senel G, Karadeniz D. Rapid eye movement sleep without atonia constitutes increased risk for neurodegenerative disorders. Acta neurologica Scandinavica. 2019;140:399-404.

[100] Cooke Jr A-ISLLLJSNLPBSHFC-BJ. Continuous positive airway pressure deepens sleep in patients with Alzheimer's disease and obstructive sleep apnea. Sleep medicine. 2009;10:1101.

[101] Cooke JR, Loredo JS, Liu L, Marler M, Corey-Bloom J, Fiorentino L, et al. Acetylcholinesterase inhibitors and sleep architecture in patients with Alzheimer's disease. Drugs & aging. 2006;23:503-11.

[102] Cooke JR, Liu L, Natarajan L, He F, Marler M, Loredo JS, et al. The effect of sleep-disordered breathing on stages of sleep in patients with Alzheimer's disease. Behavioral sleep medicine. 2006;4:219-27.

[103] Cook JD, Ferry DG, Tran KM. Sleep’s role in preventing and treating Alzheimer’s disease: Are we moving towards slow-wave assessment and enhancement? Sleep. 2020;43:1-4.

[104] Cole CS, Richards KC, Smith-Olinde L, Roberson PK, Sullivan DH. Tone-induced sleep fragmentation in persons with Alzheimer's disease: a feasibility study. Biological research for nursing. 2010;11:229-35.

[105] Chong MS, Ayalon L, Marler M, Loredo JS, Corey-Bloom J, Palmer BW, et al. Continuous positive airway pressure reduces subjective daytime sleepiness in patients with mild to moderate Alzheimer's disease with sleep disordered breathing. Journal of the American Geriatrics Society. 2006;54:777-81.

[106] Chen PC, Wu D, Chen CC, Chi NF, Kang JH, Hu CJ. Rapid eye movement sleep atonia in patients with cognitive impairment. Journal of the neurological sciences. 2011;305:34-7.

[107] Carnicelli L, Maestri M, Di Coscio E, Tognoni G, Fabbrini M, Schirru A, et al. A longitudinal study of polysomnographic variables in patients with mild cognitive impairment converting to Alzheimer's disease. Journal of sleep research. 2019;28.

[108] Boeve BF, Silber MH, Ferman TJ, Lucas JA, Parisi JE. Association of REM sleep behavior disorder and neurodegenerative disease may reflect an underlying synucleinopathy. Movement Disorders. 2001;16:622-30.

[109] Bliwise DL, Watts RL, Watts N, Rye DB, Irbe D, Hughes M. Disruptive nocturnal behavior in Parkinson's disease and Alzheimer's disease. Journal of geriatric psychiatry and neurology. 1995;8:107-10.

[110] Bliwise DL, Trotti LM, Yesavage JA, Rye DB. Periodic leg movements in sleep in elderly patients with Parkinsonism and Alzheimer's disease. European journal of neurology. 2012;19:918-23.

[111] Bliwise DL, Hughes M, McMahon PM, Kutner N. Observed sleep/wakefulness and severity of dementia in an Alzheimer's disease special care unit. Journals of Gerontology Series A: Biological Sciences & Medical Sciences. 1995;50A:M303-M6.

[112] Baumann CR, Dauvilliers Y, Mignot E, Bassetti CL. Normal CSF Hypocretin-1 (Orexin A) Levels in Dementia with Lewy Bodies Associated with Excessive Daytime Sleepiness. European neurology. 2004;52:73-6.

[113] André C, Rehel S, Kuhn E, Landeau B, Moulinet I, Touron E, et al. Association of sleep-disordered breathing with alzheimer disease biomarkers in community-dwelling older adults: a secondary analysis of a randomized clinical trial. JAMA neurology. 2020; 77(6):716-24.

[114] Allen SR, Seiler WO, Stähelin HB, Spiegel R. Seventy-two hour polygraphic and behavioral recordings of wakefulness and sleep in a hospital geriatric unit: Comparison between demented and nondemented patients. Sleep. 1987;10:143-59.

[115] Moe KE, Larsen LH, Prinz PN, Vitiello MV. Major unipolar depression and mild Alzheimer's disease: differentiation by quantitative tonic REM EEG. Electroencephalogr Clin Neurophysiol. 1993; 86(4): 238-46.

**Table S7.** Risk of bias assessment based on an adapted version of the NICE checklist in studies making comparisons between AD patients and controls.

| Author(s), date | Study addresses  an appropriate  and clearly  focused  question | The cases and  controls are  taken from  comparable  populations | The same  exclusion criteria  are used for both  cases and  controls | What was the  participation rate  for cases and  controls? | Participants and  non-participants are  compared to  establish their  similarities or  differences | Cases are  clearly defined  and differentiated  from controls | It is clearly  established that  controls are not  cases | Measures were taken  to prevent knowledge  of primary exposure  from influencing case  ascertainment. | Exposure status  is measured in a  standard, valid,  and reliable way. | The main potential  confounders are  identified and taken  into account in the  design and  analysis. |
| --- | --- | --- | --- | --- | --- | --- | --- | --- | --- | --- |
| Bonakis et al., 2014 [24] | ***√*** | ***√*** | ***√*** | ? | ? | ***√*** | ***√*** | ? | ***√*** | **×** |
| Bonanni et al., 2005 [35] | ***√*** | ***√*** | ***√*** | ? | ? | ***√*** | ***√*** | ? | ***√*** | ***√*** |
| Brunetti et al., 2020 [36] | ***√*** | ***√*** | ***√*** | ? | ? | ***√*** | ***√*** | ? | ***√*** | ***√*** |
| Chen et al., 2012 [37] | ***√*** | ***√*** | ? | ? | ? | ***√*** | ***√*** | ? | ***√*** | ***√*** |
| Dykierek et al., 1998 [38] | ***√*** | ***√*** | ? | ? | ? | ***√*** | ***√*** | ? | ***√*** | ***√*** |
| Gagnon et al., 2006 [39] | ***√*** | ***√*** | ? | ? | ? | ***√*** | ***√*** | ? | ***√*** | **×** |
| Gorgoni et al., 2016 [40] | ***√*** | ***√*** | ***√*** | ? | ? | ***√*** | ***√*** | ? | ***√*** | ***√*** |
| Hassainia et al., 1997 [41] | ***√*** | ***√*** | ***√*** | ? | ? | ***√*** | ***√*** | ? | ***√*** | ? |
| Hot et al., 2011 [42] | ***√*** | ***√*** | ? | ? | ? | ***√*** | ***√*** | ? | ***√*** | ? |
| Liguori et al., 2019 [43] | ***√*** | ***√*** | ***√*** | ? | ? | ***√*** | ***√*** | ? | ***√*** | ***√*** |
| Liguori et al., 2020 [44] | ***√*** | ***√*** | ***√*** | ? | ? | ***√*** | ***√*** | ? | ***√*** | ***√*** |
| Liu et al., 2020 [45] | ***√*** | ***√*** | ***√*** | ? | ? | ***√*** | ***√*** | ? | ***√*** | ***√*** |
| Maestri et al., 2015 [46] | ***√*** | ***√*** | ? | ? | ? | ***√*** | ***√*** | ? | ***√*** | ***√*** |
| Martin et al., 1986 [26] | ***√*** | ? | ? | ? | ? | ***√*** | ***√*** | ? | ***√*** | ***√*** |
| Montplaisir et al., 1995 [47] | ***√*** | ? | ? | ? | ? | ***√*** | ***√*** | ? | ***√*** | ? |
| Rauchs et al., 2008 [25] | ***√*** | ***√*** | ? | ? | ? | ***√*** | ***√*** | ? | ***√*** | ? |
| Reda et al., 2017 [51] | ***√*** | ***√*** | ***√*** | ? | ? | ***√*** | ***√*** | ? | ***√*** | ***√*** |
| Reynolds et al., 1985a [54] | ***√*** | ***√*** | ? | ? | ? | ***√*** | ***√*** | ? | ***√*** | ***√*** |
| Reynolds et al., 1985b [52] | ***√*** | ***√*** | ? | ? | ? | ***√*** | ***√*** | ? | ***√*** | ***√*** |
| Reynolds et al., 1988 [53] | ***√*** | **×** | ? | ? | ? | ***√*** | ***√*** | ? | ***√*** | ***√*** |
| Prinz et al., 1982a [50] | ***√*** | ***√*** | ? | ? | ? | ***√*** | ***√*** | ? | ***√*** | ? |
| Prinz et al., 1982b [49] | ***√*** | ***√*** | ? | ? | ? | ***√*** | ***√*** | ? | ***√*** | ***√*** |
| Prinz et al., 1992 [59] | ***√*** | ***√*** | ***√*** | ? | ? | ***√*** | ***√*** | ? | ***√*** | ***√*** |
| Petit et al., 1992 [48] | ***√*** | ***√*** | ? | ? | ? | ***√*** | ***√*** | ? | ***√*** | ? |
| Tseng et al., 2010 [55] | ***√*** | ***√*** | ? | ? | ? | ***√*** | ***√*** | ? | ***√*** | ? |
| Vitiello et al., 1984 [56] | ***√*** | ***√*** | ***√*** | ? | ? | ***√*** | ***√*** | ? | ***√*** | ***√*** |
| Vitiello et al., 1990 [57] | ***√*** | ***√*** | ***√*** | ? | ? | ***√*** | ***√*** | ? | ***√*** | ***√*** |
| Yin et al., 2016 [58] | ***√*** | ***√*** | ***√*** | ? | ? | ***√*** | ***√*** | ? | ***√*** | ***√*** |

Not Addressed/Reported

Poorly Addressed

Adequately Addressed

Well Addressed

***√***

***√***

**×**

?

**Table S8.** Summary of findings that were not subjected to meta-analysis.

| Study | Outcomes | Sample size | Main findings |
| --- | --- | --- | --- |
| Gorgoni et al., 2016 [40] | Sleep spindle | 15 AD;  15 controls | AD patients showed significant parietal fast spindle density decrease, which was positively associated with MMSE scores. |
| Hassainia et al., 1997 [41] | PSA | 27 AD;  25 controls | There was an increase in absolute delta and theta activities, and a decrease in absolute alpha and beta activities during REM sleep in AD patients compared with controls. Those alterations affecting particularly the parieto-temporal and frontal regions |
| Hot et al., 2011 [42] | PSA | 14 AD;  14 controls | AD patients presented faster mean theta frequency in both REM sleep and slow wave sleep (SWS). In AD patients, a correlative analysis revealed that faster theta frequency during SWS was associated with better delayed episodic recall. |
| Liu et al., 2020 [45] | Sleep spindle | 30 AD;  30 controls | AD patients had poorer spindle and KC activities compared with controls. These alterations were associated with decreased MMSE and Montreal Cognitive Assessment scores. |
| Maestri et al., 2015 [46] | CAP | 11 AD;  11 controls | AD patients showed decreased CAP rate and A1%, and increased A2% and A3% compared with controls. |
| Montplaisir et al., 1995 [47] | Sleep spindle | 10 AD;  10 controls | AD patients showed significantly decreased sleep spindle density and KC density compared with controls. |
| Petit et al., 1992 [48] | PSA | 8 AD;  8 controls | EEG slowing in AD patients was found to be much more prominent during REM sleep than during wakefulness. In addition, asymmetry on the awake EEG of Alzheimer patients was found to be even more prominent than on the REM sleep EEG. |
| Prinz et al., 1982b [49] | PSA and sleep spindle | 10 AD;  11 controls | Diffuse slowing of the EEG during waking and REM sleep was minimal in the control group, but occurred in all but one of ten AD patients, as evidenced by the 24-hour recordings. Spindle activity was poorly formed or absent in nine of the 10 AD patients, but in only one of the 11 normal controls |
| Prinz et al., 1992 [59] | PSA | 39 AD;  43 controls | The frequency spectra during tonic REM sleep revealed a significant shift towards slower wave forms in AD vs. control subjects. Beta (> 12 Hz) was reduced and theta and delta (2-8 Hz) increased in AD compared to control groups. |
| Rauchs et al., 2008 [25] | Sleep spindle | 14 AD;  14 controls | Sleep spindles were globally reduced in aging and AD. AD patients also exhibited a further decrease in fast spindles. Besides, mean intensity of fast spindles was positively correlated, in AD patients, with immediate recall performance. |
| Reda et al., 2017 [51] | K complexes | 20 AD;  20 controls | AD patients showed a significant KC density decrease compared with controls. KC density was positively correlated with MMSE score. |
| Reynolds et al., 1985b [52] | PSA | 22 AD;  24 controls | There were no significant difference in the entire spectrum of central delta activity between AD patients and controls. |

AD, Alzheimer's disease; CAP, cyclic alternating pattern; EEG, electroencephalogram; KC, K complex; MMSE, Mini-Mental State Examination; PSA, power spectral analysis; REM, rapid eye movement.

**Figure S1.** Funnel plot for meta-analysis of studies estimating the difference in total sleep time between AD patients and controls; Egger test p value=0.403.


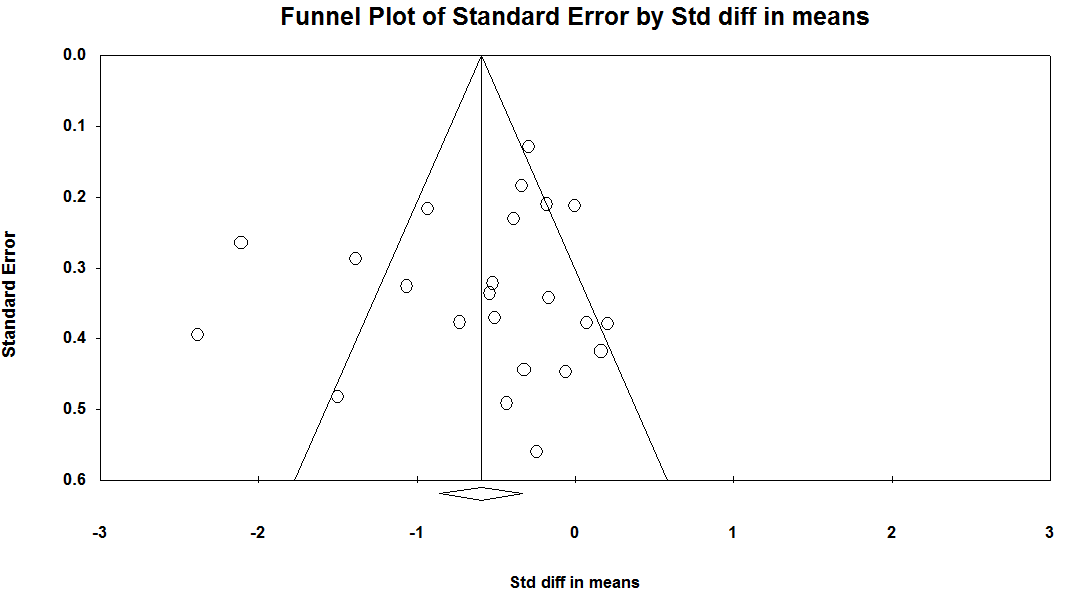


**Figure S2.** Funnel plot for meta-analysis of studies estimating the difference in wake time after sleep onset between AD patients and controls; Egger test p value=0.164.

**
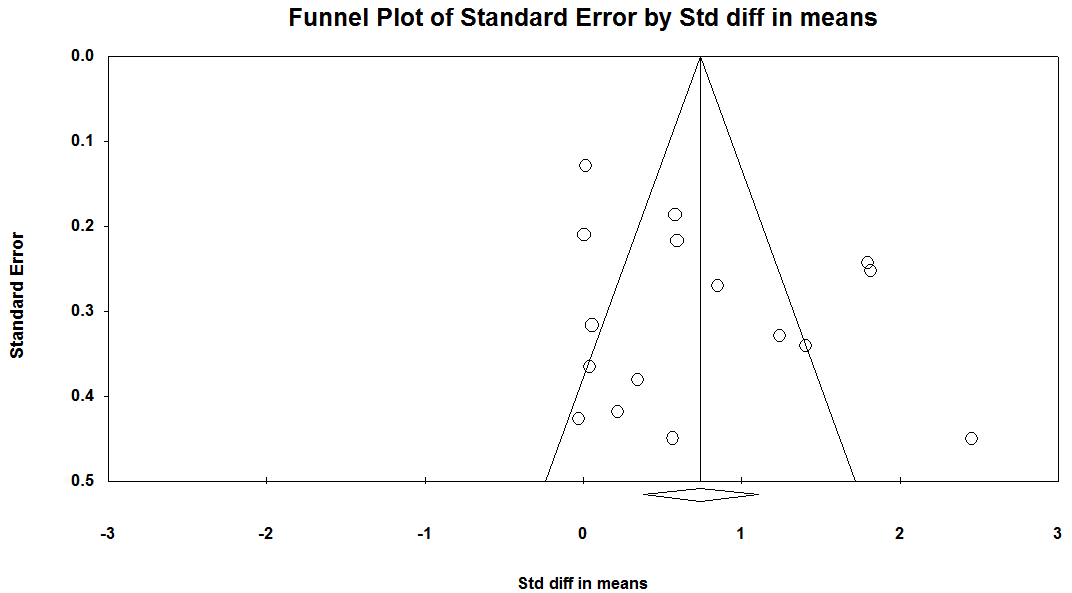
**

**Figure S3.** Funnel plot for meta-analysis of studies estimating the difference in sleep efficiency between AD patients and controls; Egger test p value=0.108.

**
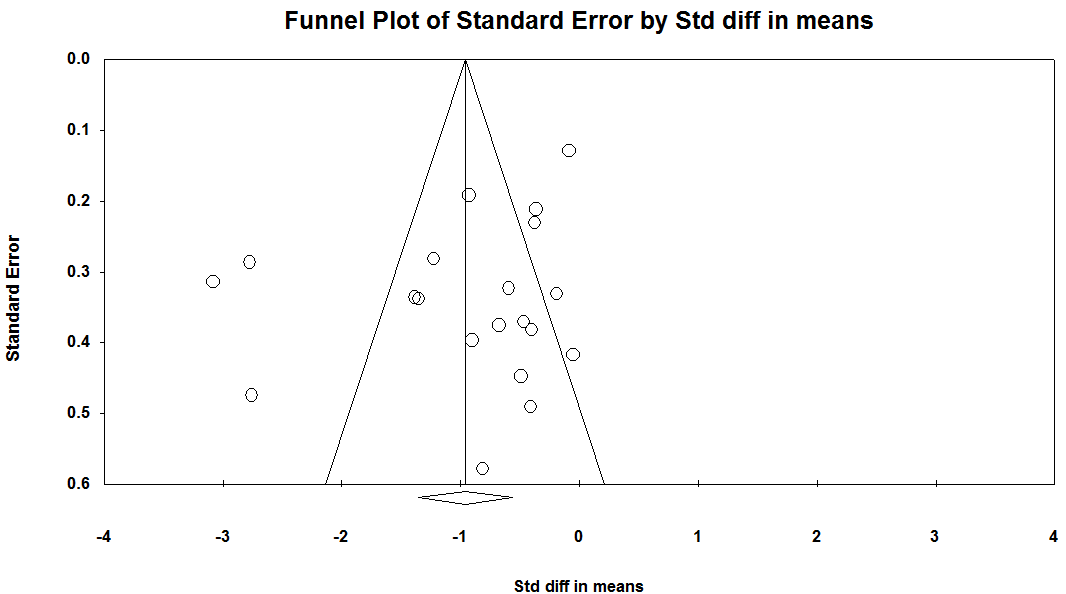
**

**Figure S4.** Funnel plot for meta-analysis of studies estimating the difference in sleep latency between AD patients and controls; Egger test p value=0.415.

**
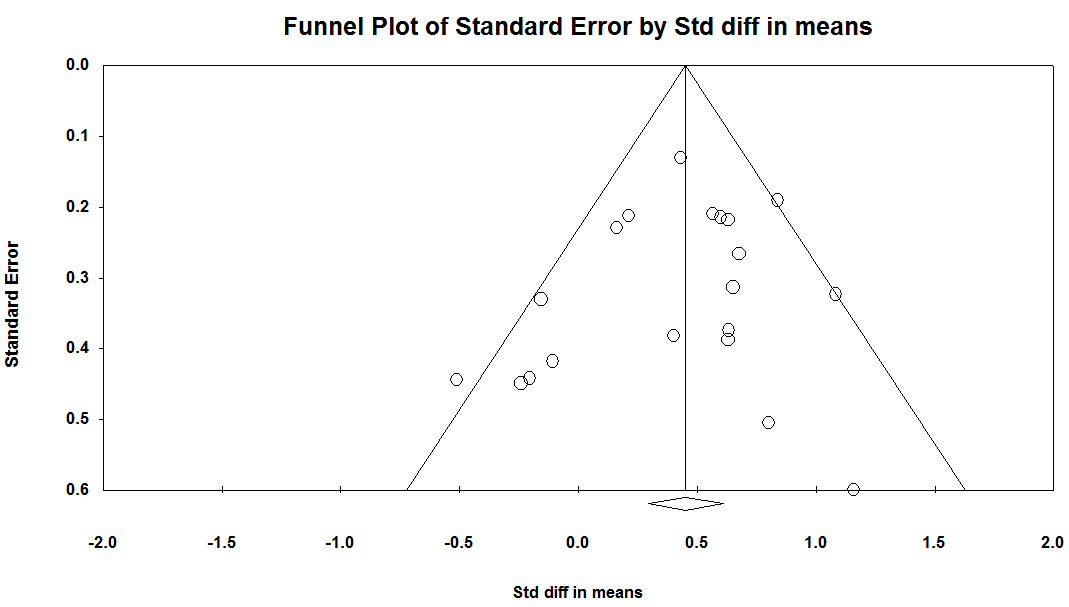
**

**Figure S5.** Funnel plot for meta-analysis of studies estimating the difference in N1 percentage between AD patients and controls; Egger test p value=0.088.


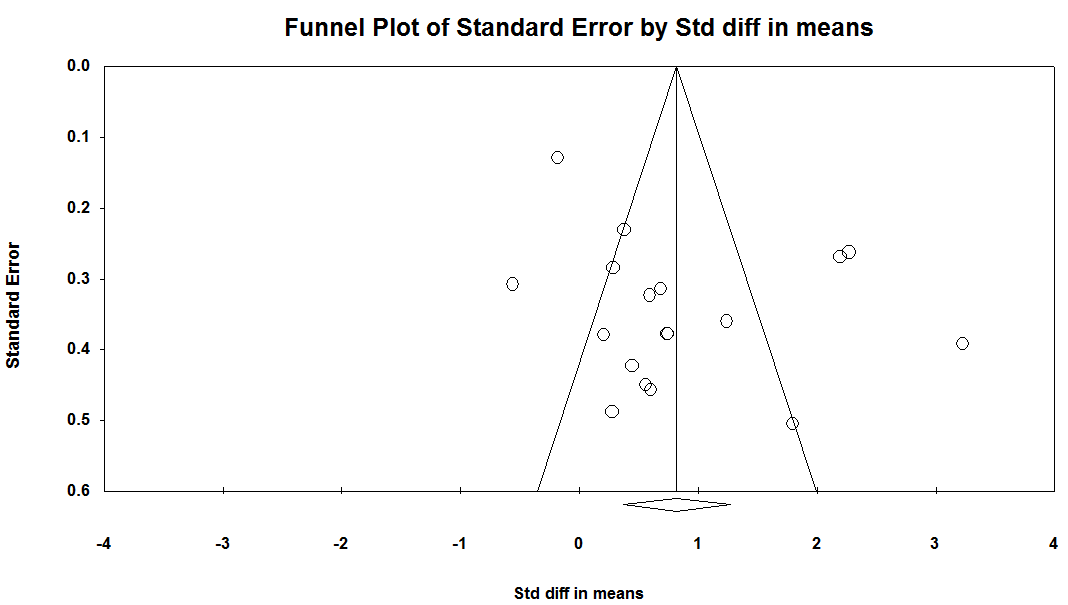


**Figure S6.** Funnel plot for meta-analysis of studies estimating the difference in N2 percentage between AD patients and controls; Egger test p value=1.000.

**
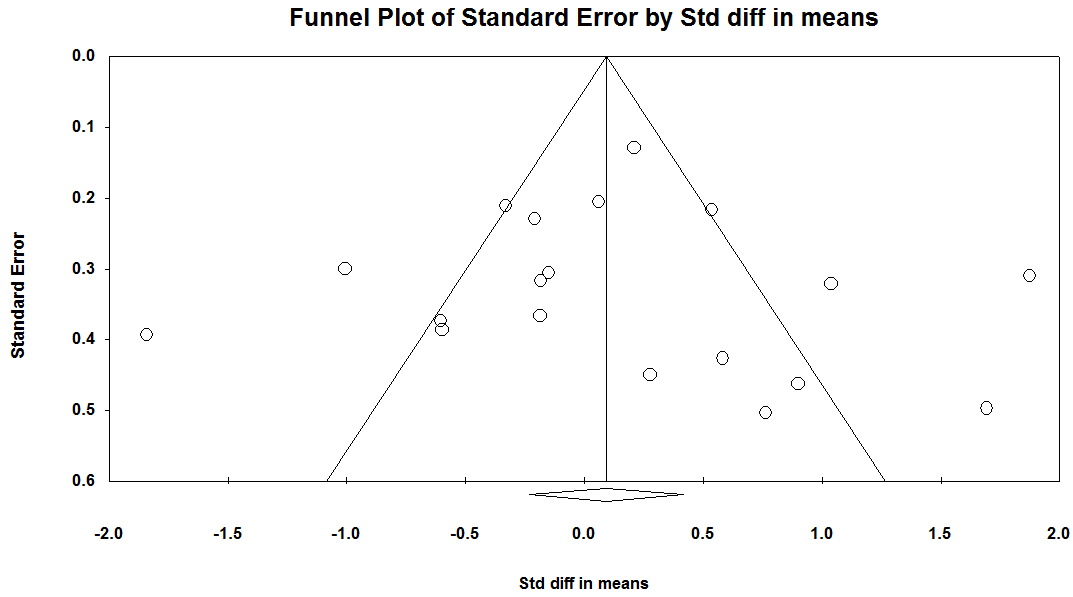
**

**Figure S7.** Funnel plot for meta-analysis of studies estimating the difference in percentage of slow wave sleep percentage between AD patients and controls; Egger test p value=0.682.


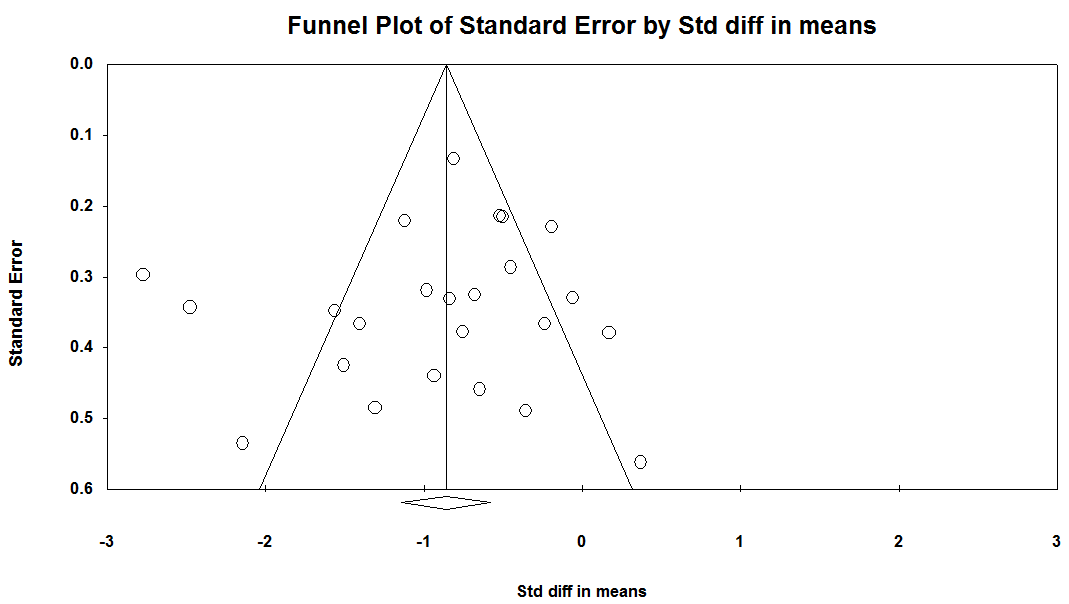


**Figure S8.** Funnel plot for meta-analysis of studies estimating the difference in percentage rapid eye movement sleep between AD patients and controls; Egger test p value=0.053.

**
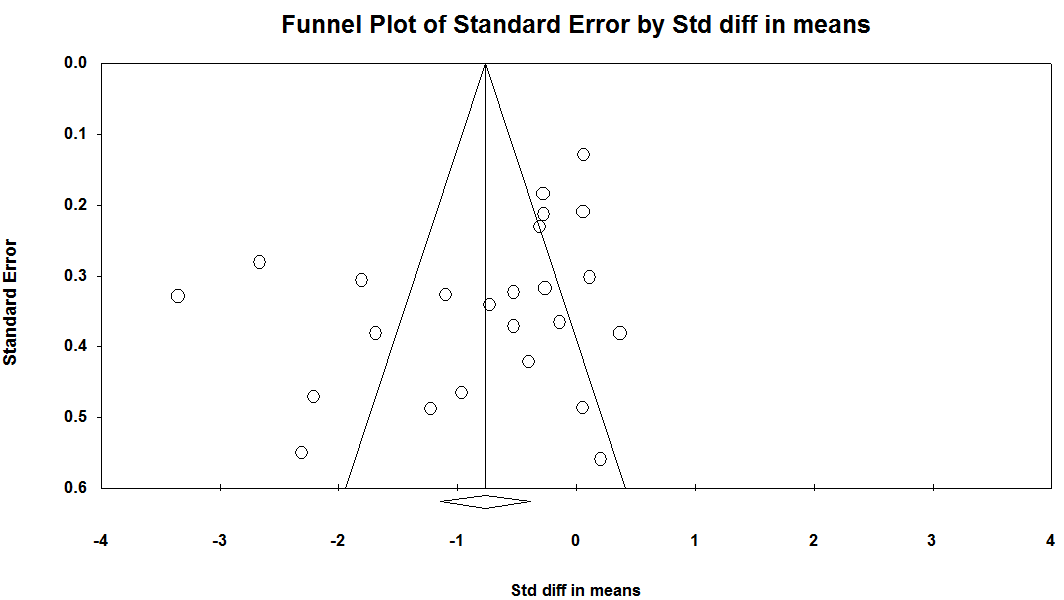
**

**Figure S9.** Funnel plot for meta-analysis of studies estimating the difference in rapid eye movement sleep latency between AD patients and controls; Egger test p value=0.490.

**
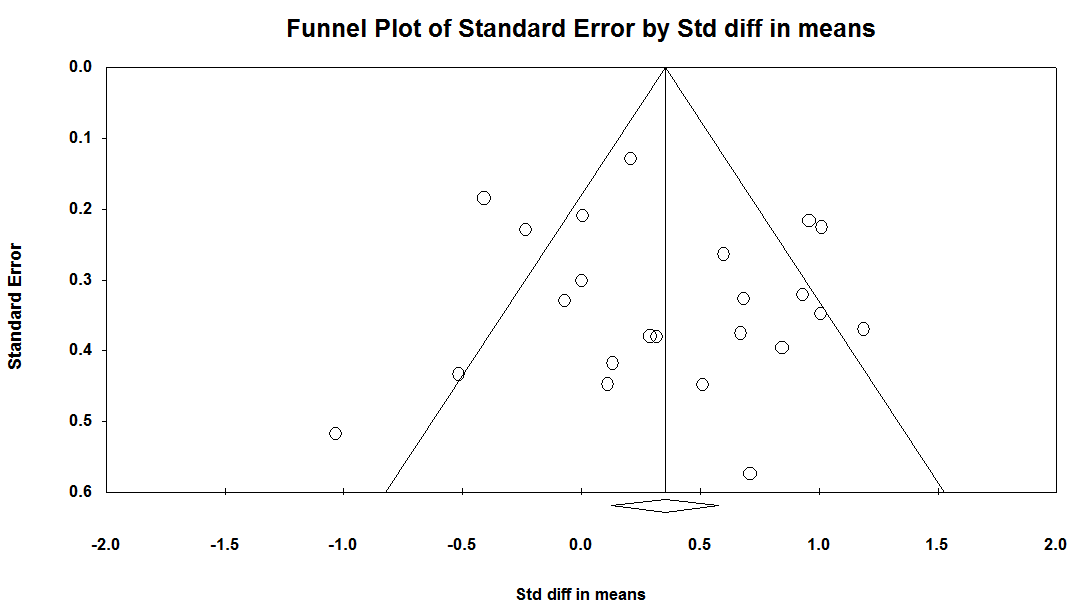
**

**Figure S10.** Funnel plot for meta-analysis of studies estimating the difference in REM density between AD patients and controls; Egger test p value=0.269.

**
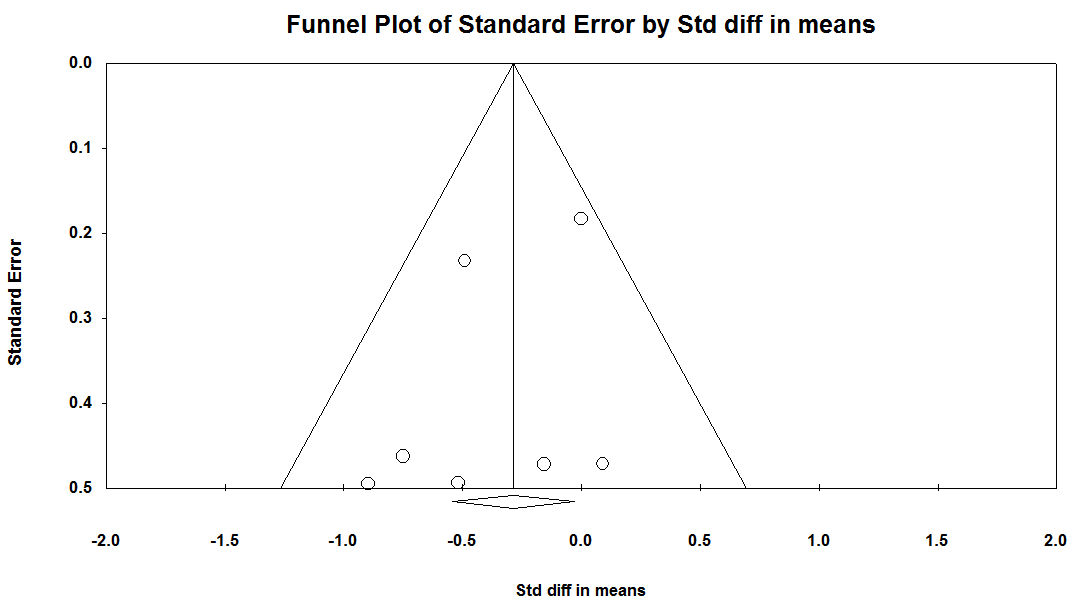
**

**Figure S11.** Funnel plot for meta-analysis of studies estimating the difference in number of awakenings between AD patients and controls; Egger test p value=0.195.


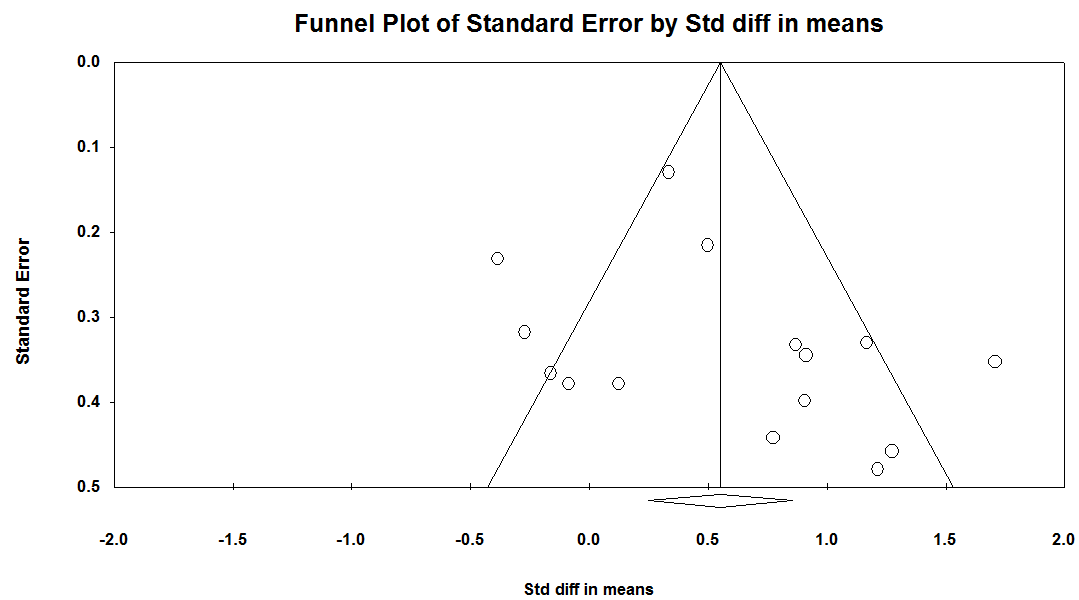

Supplement: Supplementary file 1 — Supplementary flie [file 41398_2022_1897_MOESM1_ESM.docx]
